# Reconstitution of a telomeric replicon organized by CST

**DOI:** 10.1038/s41586-022-04930-8

**Published:** 2022-07-13

**Authors:** Arthur J. Zaug, Karen J. Goodrich, Jessica J. Song, Ashley E. Sullivan, Thomas R. Cech

**Affiliations:** 1 Department of Biochemistry, University of Colorado Boulder, Boulder, CO USA; 2 BioFrontiers Institute, University of Colorado Boulder, Boulder, CO USA; 3 Howard Hughes Medical Institute, University of Colorado Boulder, Boulder, CO USA

**Keywords:** DNA, DNA synthesis

## Abstract

Telomeres, the natural ends of linear chromosomes, comprise repeat-sequence DNA and associated proteins^1^. Replication of telomeres allows continued proliferation of human stem cells and immortality of cancer cells^2^. This replication requires telomerase^3^ extension of the single-stranded DNA (ssDNA) of the telomeric G-strand ((TTAGGG)_*n*_); the synthesis of the complementary C-strand ((CCCTAA)_*n*_) is much less well characterized. The CST (CTC1–STN1–TEN1) protein complex, a DNA polymerase α-primase accessory factor^4,5^, is known to be required for telomere replication in vivo^6–9^, and the molecular analysis presented here reveals key features of its mechanism. We find that human CST uses its ssDNA-binding activity to specify the origins for telomeric C-strand synthesis by bound Polα-primase. CST-organized DNA polymerization can copy a telomeric DNA template that folds into G-quadruplex structures, but the challenges presented by this template probably contribute to telomere replication problems observed in vivo. Combining telomerase, a short telomeric ssDNA primer and CST–Polα–primase gives complete telomeric DNA replication, resulting in the same sort of ssDNA 3′ overhang found naturally on human telomeres. We conclude that the CST complex not only terminates telomerase extension^10,11^ and recruits Polα–primase to telomeric ssDNA^4,12,13^ but also orchestrates C-strand synthesis. Because replication of the telomere has features distinct from replication of the rest of the genome, targeting telomere-replication components including CST holds promise for cancer therapeutics.

## Abstract (web summary)

The Polα–primase-associated CST complex organizes telomeric C-strand DNA synthesis, and, in combination with telomerase, it carries out complete replication of the single-stranded DNA overhang found at human telomeres.

## Main

Biochemical reconstitutions using purified macromolecules have provided detailed understanding of the key steps in the central dogma of molecular biology, including DNA replication^14–16^, as well as transcription, pre-mRNA splicing and translation. The replication of telomeres, the natural ends of eukaryotic chromosomes, is now poised to be reconstituted in an equivalent manner.

Human telomeric DNA is mostly double stranded but ends in a 100- to 300-nucleotide (nt) overhang of the G-rich strand. Telomerase uses its RNA template and reverse transcriptase catalytic subunit to extend this G-strand, as first shown in the *Tetrahymena* system^3^ and subsequently in the human system^17^. In these experiments, synthetic oligonucleotides with telomeric sequences serve as primers for telomerase. Human telomerase extension terminates with the help of CST, which sequesters free primers and prevents re-initiation^10,11^.

CST is a heterotrimeric protein complex that was first identified as an accessory factor for polymerase α (Polα)–primase. CST binds single-stranded DNA (ssDNA) with some specificity for G-rich sequences^18^, which allows it to have special roles in telomere protection and replication^6–9^ and at the same time to participate in restarting stalled replication forks across the genome^9^. Sequence comparisons indicated that CST is related to RPA (replication protein A), and that relationship was confirmed by the recent cryo-electron microscopy (cryo-EM) structure of the human CST complex^19^. The cryo-EM structure identified a portion of the ssDNA-binding site within the CTC1 subunit, which allowed the design of separation-of-function mutants defective in ssDNA binding^19^.

After telomerase extends the G-strand tail at the chromosome end, Polα–primase uses this G-strand DNA as a template for complementary C-strand synthesis. Polα–primase is known to be necessary for telomere replication in vivo^20^, and it can use telomeric repeats as a template for C-strand synthesis in vitro^21,22^. The CST complex binds Polα–primase and is thought to recruit it to telomeres^4,12,13^. In fact, even before the heterotrimeric CST complex was identified in yeast, its individual subunits had been shown to be required for telomere maintenance^12,13,23,24^. The importance of CST for telomere replication was subsequently extended to plant and mammalian systems^6–9^. These pioneering studies set the stage for the reconstitution presented here.

## CST–Polα–primase uses telomeric DNA

**Extended Data Fig. 1:**
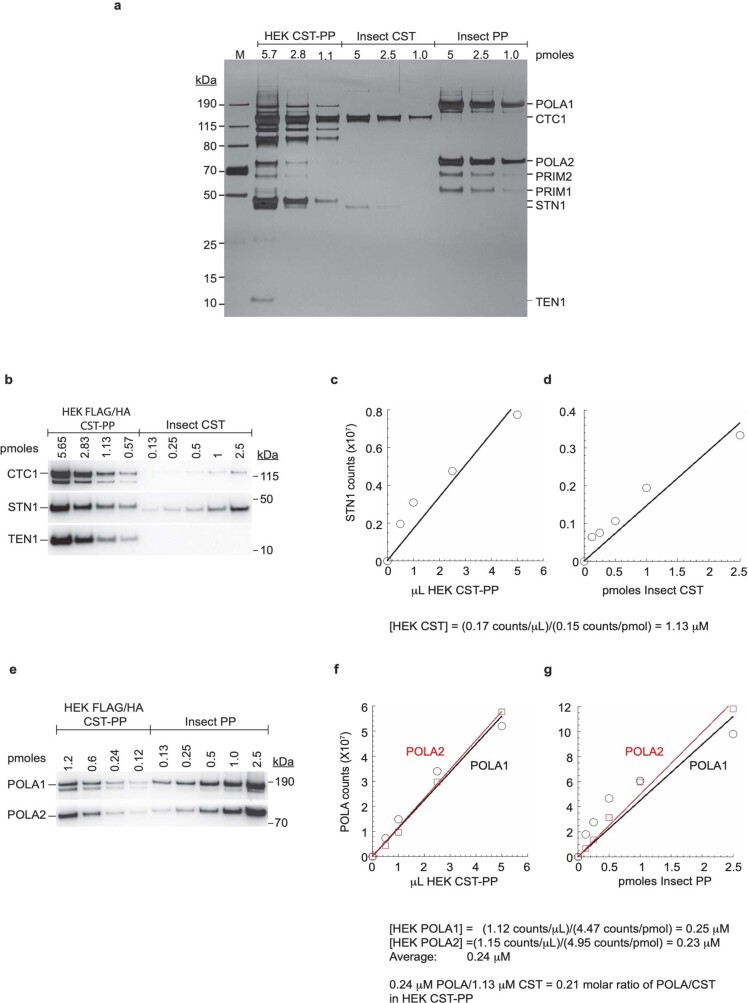
Purified CST–Polα-primase and stoichiometry of the subunits. **a**, Silver-stained SDS-PAGE of one of the HEK cell CST–Polα-primase preparations used in this study (HEK CST-PP), recombinant human CST overexpressed in insect cells (Insect CST), and recombinant human Polα-primase overexpressed in insect cells (Insect PP). M, marker proteins. The HEK cell and insect cell STN1 proteins have slightly different mobilities due to a Myc tag on the former. **b**, Western blot with antibodies to STN1 and to epitope tags on the HEK cell CTC1 and TEN1 subunits (thus no signal for CTC1 or TEN1 in the Insect CST). The HEK cell-expressed CTC1 consistently runs as a doublet. Amount of protein loaded (pmoles) is based on direct determination of protein concentration for Insect CST but is calculated from the STN1 western blots for HEK CST-PP. HEK FLAG/HA CST-PP indicates double immunopurification via FLAG and HA tags. **c** and **d**, Quantification of STN1 from the western blot gives (**c**) the phosphorimager counts of STN1 per µL for the HEK cell preparation and (**d**) the counts of STN1 per pmol for purified insect cell CST; the calculation below the graphs gives the concentration of STN1 in the HEK cell CST–Polα-primase preparation. **e**, Western blot with antibodies to the two POLA subunits. The endogenous POLA1 from HEK cells consistently runs as a doublet, likely due to processing between lys 123 and lys 124 producing a stable 165 kDa species^39^; in contrast, the POLA1 in the Polα-primase overexpressed in insect cells is mostly a single species with a minor smaller species. Amount of protein loaded (pmoles) is based on direct determination of protein concentration for Insect PP but is calculated from the POLA western blots for HEK CST-PP. **f**, **g**, Quantification of POLA1 and POLA2 from the western blot gives (**f**) the counts of POLA1 and POLA2 per µL of the HEK cell preparation and (**g**) the counts per pmol for the purified insect cell Polα-primase; the calculation below the graphs gives the concentration of POLA in the HEK cell CST–Polα-primase preparation. Dividing the concentration of POLA from **f** and **g** by the concentration of CST from **c** and **d** gives the stoichiometry of 0.21 POLA per CST in the HEK cell preparation. These results were consistent between two biological replicates of the entire set of experiments. For gel source data, see Supplementary Fig. 1 Source Data

When we express recombinant human CST in HEK293T cells, the affinity-purified CST also contains the four subunits of Polα–primase^11^. Because Polα-primase is not overexpressed, it represents the endogenous enzyme. The co-purification of Polα–primase with CST is consistent with the known binding interaction^4,12,13^. Quantitative analysis revealed that Polα–primase was substoichiometric, present at 21% the level of the CST heterotrimer (Extended Data Fig. 1).

**Fig. 1:**
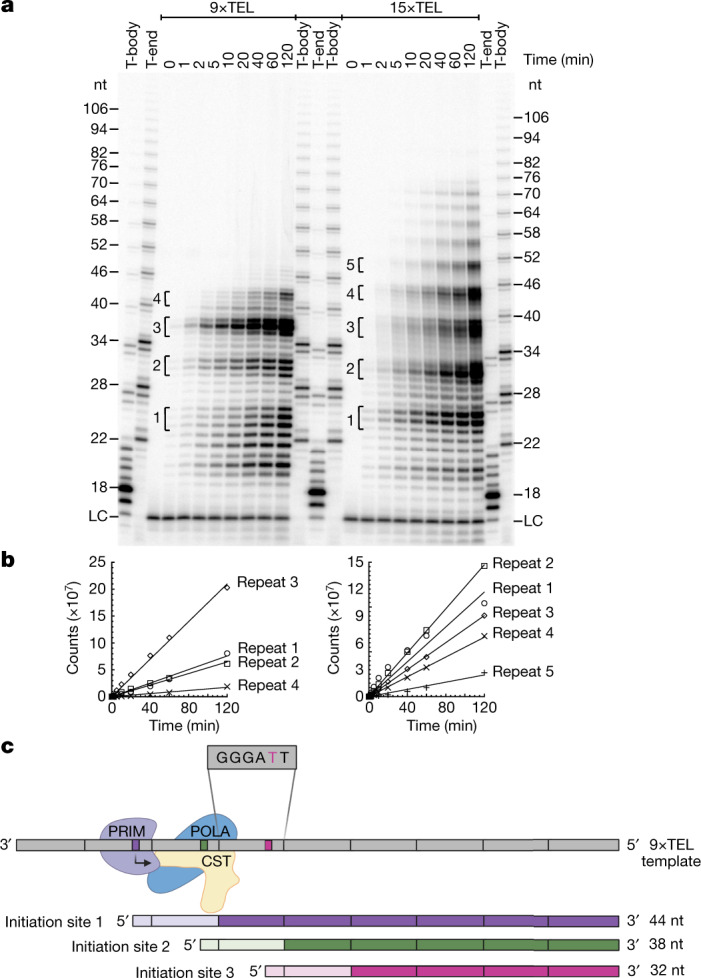
CST–Polα–primase uses telomeric repeats as origins of priming and replication. **a**, Time courses of C-strand synthesis on telomeric DNA templates, with products labelled with [α-^32^P]dCTP. Size markers are telomerase reaction products with end-labelled primer (T-end, 5′-phosphate) and body-labelled products (T-body, 5′-hydroxyl) as described in Methods. LC, labelled oligonucleotide loading control. **b**, Quantification of C-strand synthesis products in the experiment directly above. **c**, Model explaining the observed 6-nt ladders of C-strand reaction products. CST–Polα–primase binds the template (grey) at different sets of telomeric repeats, such that runoff synthesis gives products in 6-nt increments. The binding site shown at the top (initiation site 1) gives the purple C-strand product, while initiation at sites 2 and 3 gives the green and red products, respectively. In each case, the RNA primer is indicated by a lighter shade and telomeric repeats are indicated by vertical bars. The location of CST relative to Polα–primase and the template DNA is taken from the recent cryo-EM structure^32^. For gel source data, see Supplementary Fig. 1. Source Data

**Extended Data Fig. 2:**
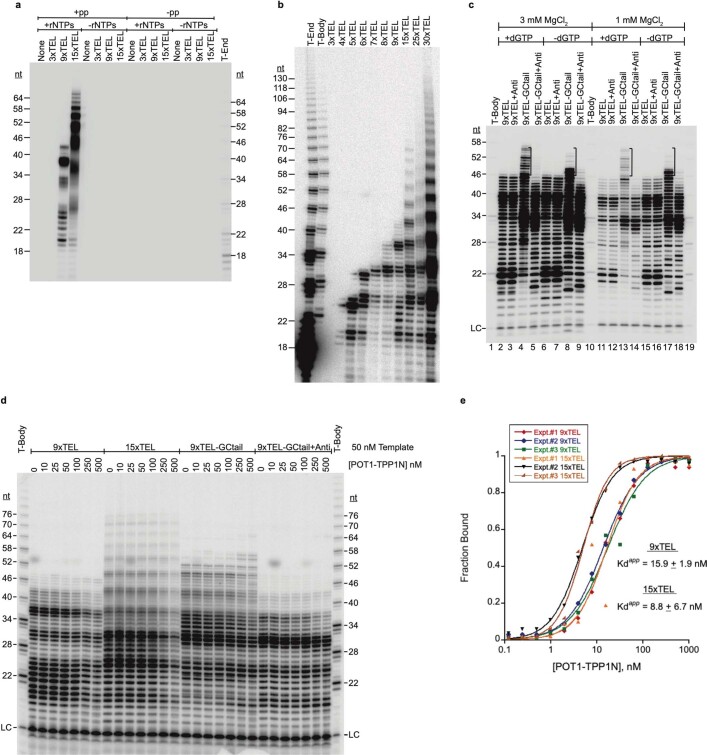
Validation of the C-strand synthesis reaction on telomeric DNA templates. **a**, Product formation requires DNA template, CST–Polα-primase, and ribonucleotides (rNTPs). +pp: CST–Polα-primase. -pp: CST with Polα-primase removed by 300 mM NaCl elution. DNA templates are None, or 100 nM 3xTEL, 9xTEL and 15xTEL. Products labeled with α-^32^P-dCTP. Nucleotide (nt) sizes are based on telomerase reaction with a 5’-end labelled primer (T-End). **b**, Longer DNA templates give larger ladders of C-strand products. Templates consist of the number of telomeric repeats indicated. 3xTEL and 4xTEL do not support DNA synthesis under these conditions. Products labeled with α-^32^P-dCTP. T-Body is a marker lane of body-labelled telomerase products, which run about one nt slower than the T-End products; the T-End products have a 5’-phosphate which increases their electrophoretic mobility. **c**, Adding a 10-nt tail to the 5’ end of a 9xTEL template confirms that at least some products runoff the end of the template, and adding an antisense oligonucleotide to pair with the 10-nt tail shows that C-strand synthesis stops at double-stranded DNA. 'GC tail' is 5’-CGCCGCCGCC, followed by (TTAGGG)_9_, and the 'anti' oligo is 5’-CTAAGGCGGCGGCG, designed to pair with the GC tail and the first four nt of the adjacent telomeric repeat. Products labelled with α-^32^P-dATP*.* Reactions performed at two MgCl_2_ concentrations as indicated. Reactions +dGTP allowed DNA synthesis to use the GC tail as template (brackets), while the sets of lanes without dGTP still gave some synthesis because of misincorporation. Addition of the anti oligo (lanes 5, 9, 14, 18) inhibited use of the GC tail as template; as a control (lanes 3, 7, 12, 16), the anti oligo had no effect with the 9xTEL template, which contains no complementary sequence. LC, loading control. **d**, POT1-TPP1N bound to telomeric DNA templates does not prevent CST–Polα-primase action but inhibits partially at high molar excess of POT1-TPP1N/template. Four DNA templates at 50 nM preincubated for 30 min at 30 °C with indicated concentrations of POT1-TPP1N prior to 1 h CST–Polα-primase reaction at 30 °C. Comparing 9xTEL-GC tail without and with the antisense oligonucleotide (anti) that binds the GC tail again confirms that C-strand synthesis stops when it encounters dsDNA. LC, loading control. **e**, POT1-TPP1N binding to trace amounts of radiolabelled 9xTEL and 15xTEL DNA as determined by electrophoretic mobility shift assay (EMSA). Binding for 30 min at 30 °C in C-strand synthesis buffer. *K*_d_^app^ values ± SD (n = 3 independent binding curves). For gel source data, see Supplementary Fig. 1 Source Data

To test the activity of this human cell CST–Polα–primase, we prepared ssDNA templates corresponding to telomerase extension products (where ‘*R*×TEL’ represents *R* repeats of TTAGGG). When CST–Polα–primase was incubated with 9×TEL and 15×TEL templates, ladders of products with a periodicity of ~6 nt were synthesized (Fig. 1a,b). Formation of products was dependent on the CST having Polα–primase bound and on inclusion of both ribo- and deoxyribonucleotides in the reaction (Extended Data Fig. 2a). Each ladder extended to a length less than that of the template, consistent with the C-strand products initiating at various telomeric repeats and running off the 5′ end of the template (Fig. 1c). Note that the most prominent product with 9×TEL as template (‘repeat 3’, ~38 nt) appears to initiate within the third telomeric repeat from the 3′ end, but a minor ~44-nt product (‘repeat 4’) has a length indicating initiation within the second telomeric repeat. The shortest purely telomeric DNA that had robust template activity was 5×TEL (Extended Data Fig. 2b). The site of RNA priming varied somewhat among templates, perhaps determined by which telomeric repeats form G-quadruplex (GQ) structures.

The model that C-strand synthesis initiates at each telomeric repeat and continues to the end of the template was supported by two additional tests. First, quantification of the time course showed that all products increased with the same kinetics (Fig. 1b), indicating that the shorter products were not intermediates that could be chased into the longer products. Second, adding a non-telomeric 10-nt extension to the 5′ end of a 9×TEL template gave 10-nt-longer runoff products (Extended Data Fig. 2c). When we added an antisense oligonucleotide complementary to the 10-nt tail, formation of these extended products was inhibited (Extended Data Fig. 2c), indicating that CST–Polα-primase stopped when it encountered double-stranded DNA (dsDNA).

Natural telomeric ssDNA would be bound by the shelterin protein heterodimer POT1–TPP1 (ref. ^1^). We therefore tested C-strand synthesis on various telomeric DNA templates bound to POT1–TPP1N. (TPP1N includes the POT1-binding and telomerase-activating domains of TPP1; ref. ^1^). At a concentration equimolar to the template, POT1–TPP1N had no effect on C-strand synthesis (Extended Data Fig. 2d), even though most template strands had POT1–TPP1N bound at this 50 nM concentration (Extended Data Fig. 2e). Our interpretation is that POT1–TPP1N binds to any of multiple positions along a telomeric DNA template, so CST–Polα–primase simply finds an unoccupied telomeric repeat to bind and initiate DNA synthesis. Ten-fold excess POT1–TPP1N, which begins to coat the DNA template, resulted in substantial inhibition of C-strand synthesis as expected (Extended Data Fig. 2d).

A striking feature of the C-strand synthesis is the 6-nt ladder of products that looks superficially like the 6-nt repeats produced by telomerase. Yet our data indicate that the mechanisms are very different. With telomerase, there is a single point of initiation (the 3′ end of the primer) and elongation can terminate or pause after each repeat, where telomerase must translocate to continue. With CST–Polα–primase, on the other hand, synthesis initiates at multiple sites, phased by the telomeric repeats, and then extends as far as the end of the template (Fig. 1c).

## Minimum origin requirement

**Fig. 2:**
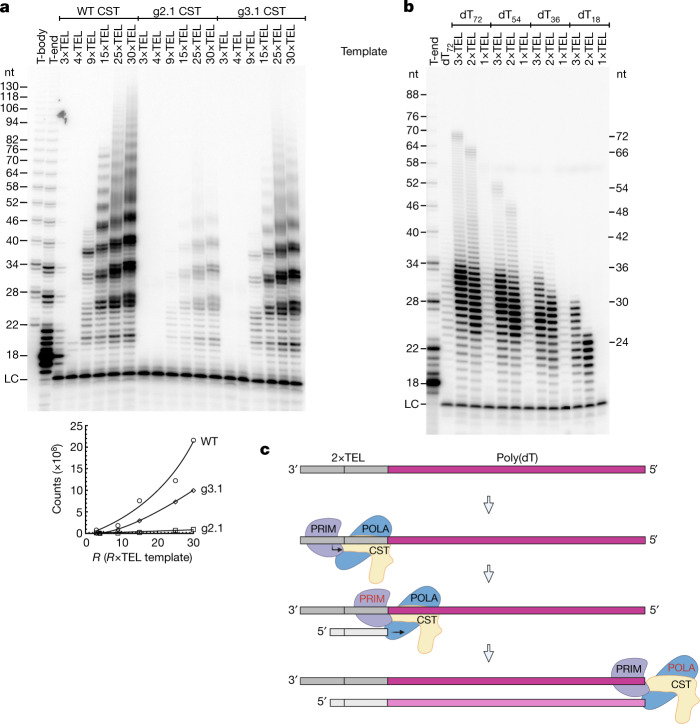
C-strand synthesis requires DNA binding by CST–Polα–primase, and a CST–Polα–primase-binding site greatly enhances replication of a poly(dT) template. **a**, C-strand synthesis for 1 h with DNA templates (100 nM) comprising increasing numbers of telomeric repeats catalysed by WT and two different DNA-binding mutants of CST–Polα–primase (20 nM). The apparent products in the third lane (3×TEL template) are spillover from the adjacent marker lane; repeat experiments confirmed that no product is formed. LC, loading control. Below, quantification of the experiment in **a**. Total incorporation was normalized to the loading control as a function of the number of telomeric repeats in the template. **b**, Replication products of CST–Polα–primase on a poly(dT) template, dT_72_, and on templates with various numbers of telomeric repeats added to the 3′ end of the poly(dT) sequence. **c**, Model for the initiation of CST–Polα–primase at the telomeric repeats and extension along the template. Whether CST remains bound to Polα–primase during extension is unknown. The length of the rose-coloured C-strand is limited by the template length (shown here) or, for longer templates, by the intrinsic processivity of the enzyme. For gel source data, see Supplementary Fig. 1. Source Data

If telomeric repeats define origins of priming and replication due to the DNA-binding specificity of CST, then C-strand synthesis should require the DNA-binding activity of CST. We tested this hypothesis with two DNA-binding mutants of the CTC1 subunit of CST, both of which still bind Polα–primase^11^. Each mutant has groups (‘g’) of two or three amino acids mutated. The g2.1 mutant of CST (32-fold-lower affinity for 3×TEL DNA) had <5% C-strand synthesis activity with multiple templates, while the less impaired g3.1 mutant (15-fold-lower affinity) had about half the activity of wild-type (WT) CST (Fig. 2a). Thus, DNA binding by CST is important for CST–Polα-primase action.

**Extended Data Fig. 3:**
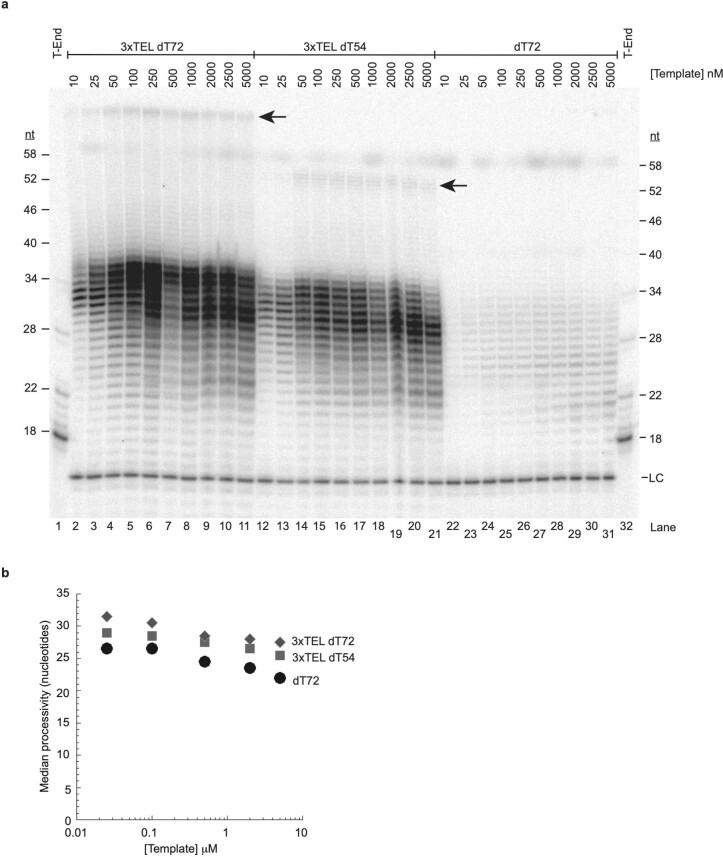
Processivity of CST–Polα-primase on poly(dT) templates. **a**, Reactions as in Fig. 2b but with increasing concentrations of the indicated templates. Products labelled with α-^32^P-dATP. LC, loading control. Arrows, rare extension to the ends of these templates. Note that the templates containing 3xTEL give much more product than poly(dT), but the length of the products (which at high [template] is limited by processivity) is similar for all templates. **b**, The intensity of each extension product (that is, the counts in each band of a lane in **a**) was divided by the number of As in that product and the median length was calculated. Diamonds, 3xTELdT72. Squares, 3xTELdT54. Circles, dT72. The observation that the median extension is largely independent of the template DNA concentration means that extension is limited by the processivity of the enzyme on that template. For gel source data, see Supplementary Fig. 1 Source Data

Independent evidence for the importance of DNA binding by CST for Polα–primase recruitment came from testing poly(dT), which is used classically as a template for Polα–primase but does not bind well to CST^18^. Poly(dT) showed extremely low activity with CST–Polα–primase (see dT_72_ in Fig. 2b). According to our model, in which TEL repeats act as origins of replication for CST–Polα–primase, adding a CST–binding sequence to the 3′ ends of poly(dT) templates should increase their use. Indeed, adding a 3×TEL sequence increased activity by 10- to 15-fold (range of four independent experiments; for example, 3×TEL-dT_72_ and 3×TEL-dT_54_ in Fig. 2b). Thus, CST can recruit Polα–primase to telomeric DNA repeats and greatly increase the template activity of adjacent non-telomeric sequences. On the other hand, the processivity of CST–Polα–primase was not much greater on the 3×TEL-poly(dT) templates than on poly(dT), suggesting that CST enhances C-strand synthesis at the level of initiation rather than elongation (Extended Data Fig. 3).

We next asked whether fewer than three TEL repeats might suffice for an origin of replication. We found 2×TEL to be just as good an origin as 3×TEL (Fig. 2b). Because the TEL sequence begins with two T’s, 2×TEL provides just 10 nt before the poly(dT) template. By contrast, 1×TEL was completely inactive as an origin, as indicated by the 1×TEL-dT_72_ and 1×TEL-dT_54_ templates giving the same faint signal as the dT_72_ control. The longest products formed with 2×TEL-dT_18_ and 3×TEL-dT_18_ were ~24 and ~30 nt, respectively (Fig. 2b), indicating that CST–Polα–primase initiated primer synthesis in the 3′-terminal TEL repeat (Fig. 2c). For the templates containing longer stretches of dT, most products were 24–32 nt, limited by the low inherent processivity of the enzyme^25^ (Extended Data Fig. 3).

**Extended Data Fig. 4:**
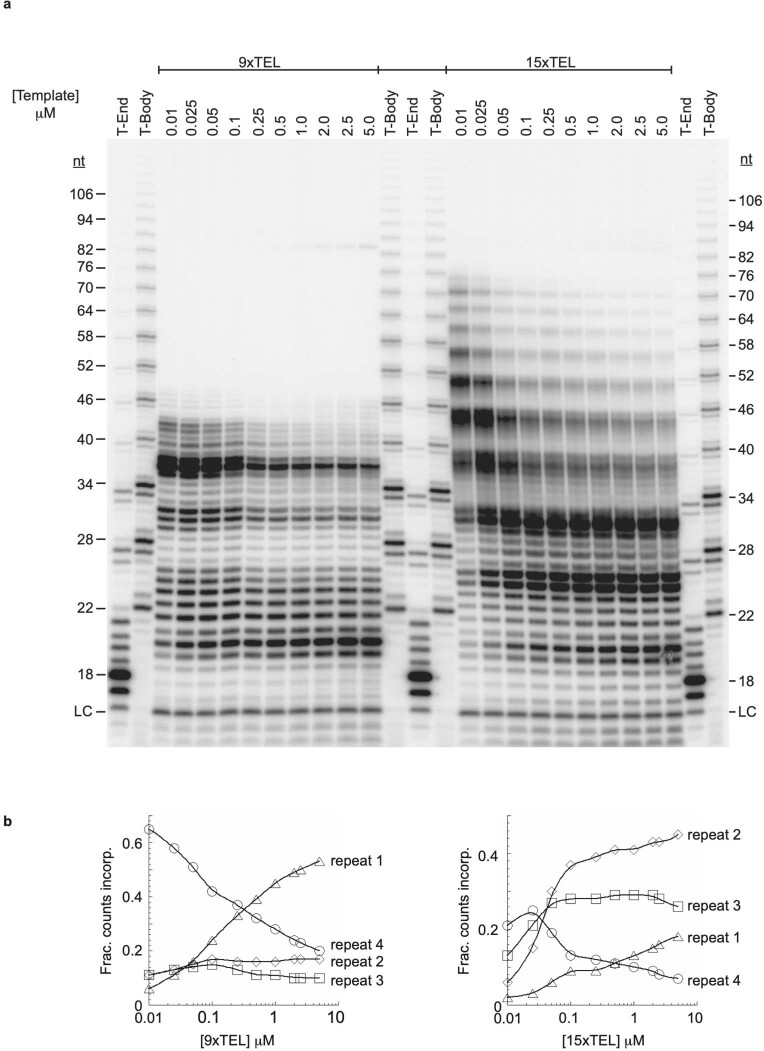
Processivity of CST–Polα-primase on 9xTEL and 15xTEL templates; increasing DNA template concentration distinguishes processive C-strand synthesis from distributive re-priming. **a**, Reaction of 25 nM CST–Polα-primase with increasing concentrations of 9xTEL or 15xTEL DNA template. **b**, Quantification of groups of extension products ('repeats') from **a**. Numbering of repeats is the same as in Fig. 1a. Increase of counts of repeat 1 products with increasing template concentration is interpreted as distributive re-priming, whereas the fraction of repeat 4 or 5 products that persists at high template concentration (1 µM template is 40-fold excess over concentration of CST–Polα-primase) is interpreted as processive extension. Repeat 4 is synthesized by approximately 70% re-priming and 30% processive synthesis. Products longer than repeat 4 are synthesized more by re-priming. Products shorter than repeat 4 persist at high [template], so they are due to processive extension from multiple initiation sites to the end of the template. For gel source data, see Supplementary Fig. 1 Source Data

It initially appeared that CST–Polα–primase was more highly processive on the 9×TEL and 15×TEL templates, on the basis of the appearance of longer C-strand products (Fig. 1a). However, multiple rounds of distributive extension involving repriming by Polα–primase represent an alternative pathway to obtain long extension products. To distinguish between these mechanisms, we evaluated processivity by performing reactions at increasing DNA template concentrations, such that any distributive extension would occur on a new template and reduce the product size distribution (Extended Data Fig. 4). The partial reduction of long products at high template concentrations revealed that both repriming and processive extension contributed to these products (>32 nt). It is not clear whether such repriming would occur in vivo, where the products made by Polα–primase are transferred to DNA polymerase 𝛿 for high-fidelity replication^20^.

## Initiation with an RNA primer

**Extended Data Fig. 5:**
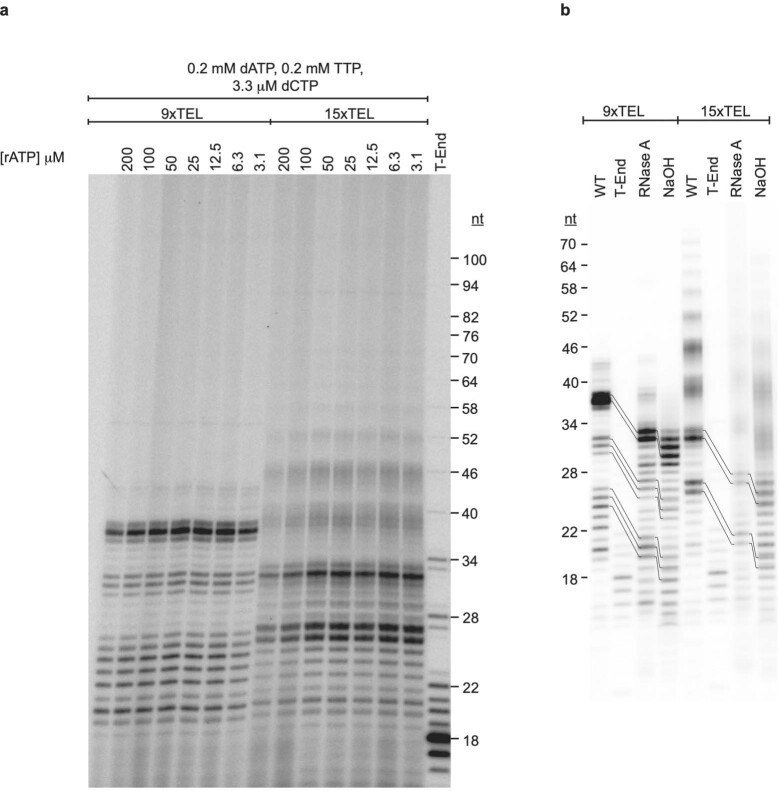
RNA primers initiate with ATP and are approximately 8 nt long. **a**, Reactions with γ-^32^P-ATP (trace concentration) as the only labelled nucleotide, with increasing concentrations of unlabelled ATP as indicated. Label incorporation decreases at 100 and 200 µM rATP, because above *K*_*m*_, dilution of the radiolabelled rATP is no longer compensated by increased reaction velocity. **b**, Hydrolysis of the RNA primer with NaOH or RNase A shortens the dC-labelled C-strand. WT, wild-type CST–Polα-primase, T-End, 5’-end labeled telomerase reaction products as size markers. RNase A, reaction products from lane WT were boiled and then treated with RNase A. NaOH, reaction products from lane WT were treated with NaOH. The 8-nt reduction in dC-labeled product size upon NaOH treatment indicates that the C-strands initiate with pppAACCCUAA/dCdCdC…, where the RNA primer is underlined and the slash indicates the 3’-most site of hydrolysis. RNase A, which cleaves after pyrimidines, would then cleave as follows: pppAACCU/AAdCdCdC…. This would result in the RNase A products being two nt longer than the NaOH products, as observed. The lines connecting the bands on the gel indicate the reduction in product size upon NaOH or RNase A treatment. For gel source data, see Supplementary Fig. 1.

**Extended Data Fig. 6:**
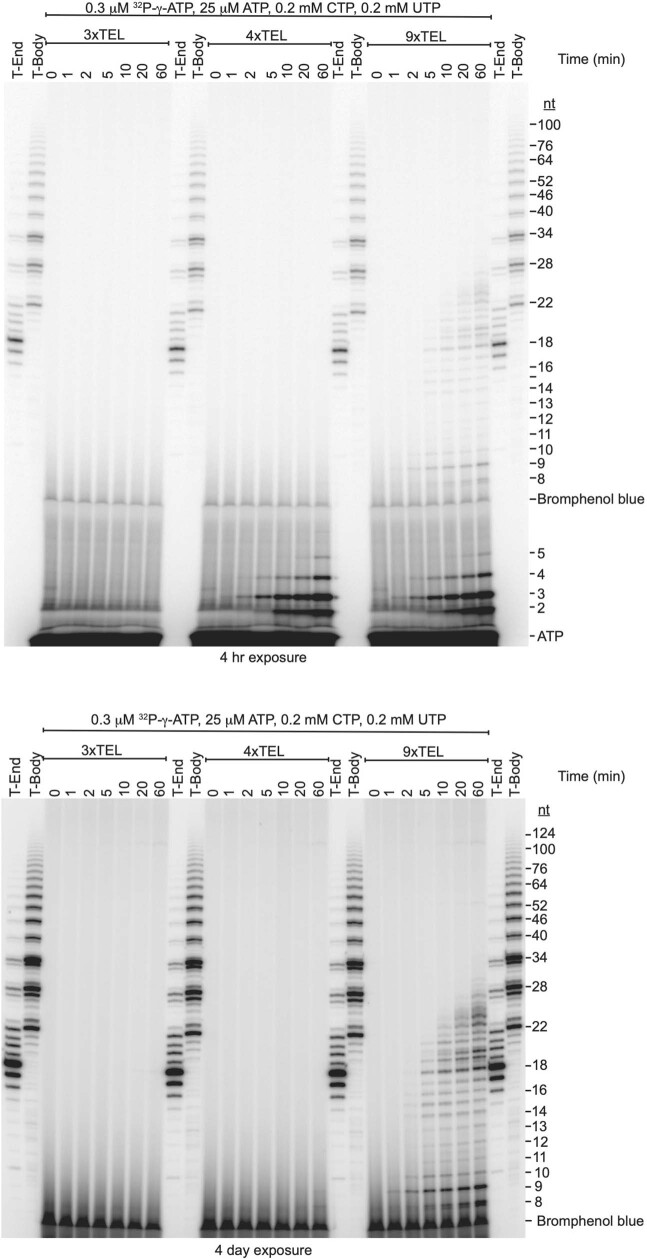
RNA primers synthesized by CST–Polα-primase on three different DNA templates, directly visualized by omitting deoxynucleotides from the reaction. Left half, production of short RNA oligonucleotides initiating with pppA on the 4xTEL and 9xTEL DNA templates. Their mobilities are consistent with 2, 3 and 4 nt, as indicated, although these sizes have not been independently confirmed. These products may represent abortive initiation by primase^27^. Right half, the bottom of the gel containing the unincorporated ^32^P-ATP was cut off to allow a longer exposure of the larger products. The 9xTEL template allowed synthesis of RNA primers of length 8 and 9 nt; size estimates have ± 1 nt uncertainty because they are based on the telomerase size markers, which have a different 5’ end and different base composition. There are also some longer products formed (14 – 27 nt), presumably indicating some ability of Polα to incorporate ribonucleotides when there are no dNTPs in the reaction. The 4xTEL template, which supported synthesis of the putative abortive initiation products, was too short to allow synthesis of the 8–9 nt primers. For gel source data, see Supplementary Fig. 1.

Because the CST-directed C-strand synthesis had several unique features, we tested whether it still retained the hallmarks of a Polα–primase reaction. Classically, the primase subunit initiates with ATP, synthesizes a 7- to 10-nt RNA primer and then hands the primer to Polα, which extends it with deoxynucleotides^21,26,27^. Four experiments showed that the C-strands initiated with an RNA primer. First, C-strand synthesis required ribonucleotides in addition to deoxyribonucleotides (Extended Data Fig. 2a). Second, with [γ-^32^P]ATP as the only label, products were formed on the 9×TEL and 15×TEL templates, similar to those labelled with [α-^32^P]dCTP (Extended Data Fig. 5a). Because the 5′-terminal label is retained on the products, synthesis must initiate with ATP. Third, NaOH treatment to hydrolyse RNA shifted the ladders of products down by ~8 nt, indicating the approximate length of the RNA primer; treatment with RNase A caused a slightly smaller shift, as expected given its sequence specificity for pyrimidines (Extended Data Fig. 5b). Fourth, reactions in the absence of deoxynucleotides allowed direct visualiztion of approximately 8- to 9-nt RNA primers (Extended Data Fig. 6). The 4×TEL template, which was too short to support DNA synthesis, was able to initiate RNA primer synthesis, whereas the 3×TEL template was not (Extended Data Fig. 6).

## Problematic GQs

**Fig. 3:**
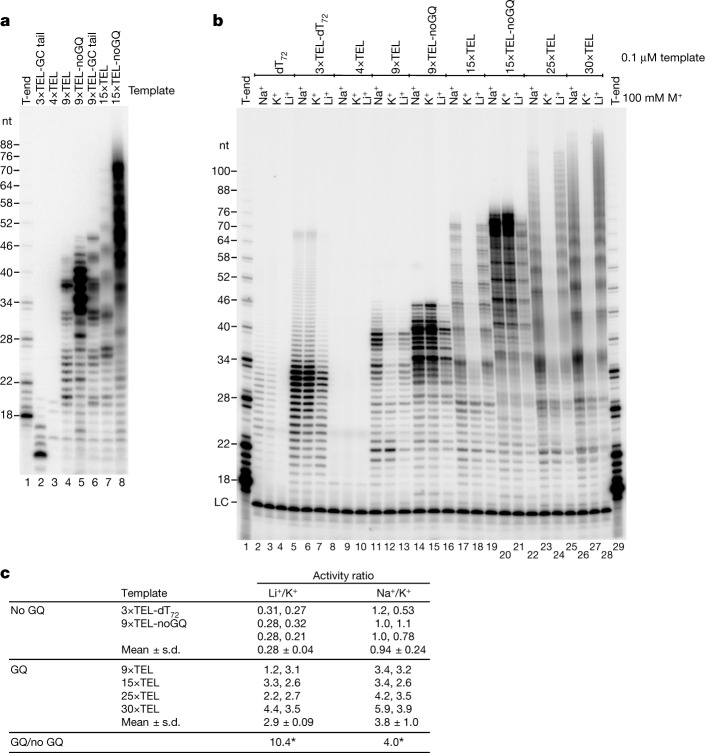
C-strand synthesis overcomes GQ structures in the template DNA. **a**, Mutating the telomeric repeat sequence (noGQ) greatly increases C-strand synthesis (compare lane 4 with lane 5 and lane 7 with lane 8). The GC tail templates have 10-nt GC sequences added on their 5′ ends to test whether the C-strand synthesis proceeds to the end of the template (compare lanes 4 and 6). Although 3×TEL is inactive, adding the 10-nt tail to 3×TEL provides a template for C-strand synthesis (lane 2). **b**, C-strand synthesis when GQ structures are destabilized (LiCl) or stabilized (KCl). NaCl is an intermediate condition. CST–Polα–primase at 25 nM; DNA templates at 100 nM. **c**, Activity with 100 mM LiCl relative to that in 100 mM KCl or activity with 100 mM NaCl relative to that with 100 mM KCl; quantification of two experiments including the one in **b**. *P < 0.0001, two-tailed *t*-test; *n* = 6 independent experiments for the noGQ templates and *n* = 8 independent experiments for the GQ templates. For gel source data, see Supplementary Fig. 1.

**Extended Data Fig. 7:**
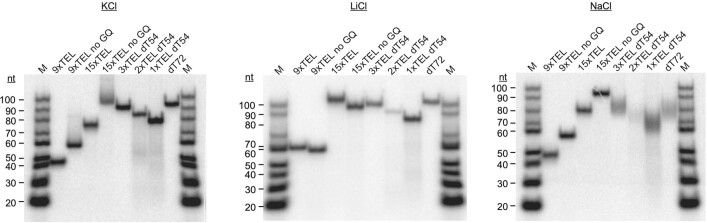
Testing if DNA templates fold into G-quadruplex structures. Template DNAs were 5’-end labelled with ^32^P, incubated at 30 °C in buffer containing 100 mM of the indicated salt, and then subjected to electrophoresis in a polyacrylamide gel containing 100 mM of the same salt in both the gel and the running buffer. Gels were run in a warm room at 30 °C, the same temperature as the DNA replication reactions. In LiCl, oligonucleotides ran largely according to their length; there are small differences due to base composition (that is, the 72-nt dT72 has an electrophoretic mobility similar to that of the 90-nt 15xTEL and 15xTEL-noGQ oligos). In KCl and NaCl, two of the oligos – 9xTEL and 15xTEL – showed anomalously fast mobility due to G-quadruplex formation. M, DNA length standards purchased from IDT and then 5’-end labelled. For gel source data, see Supplementary Fig. 1.

**Extended Data Fig. 8:**
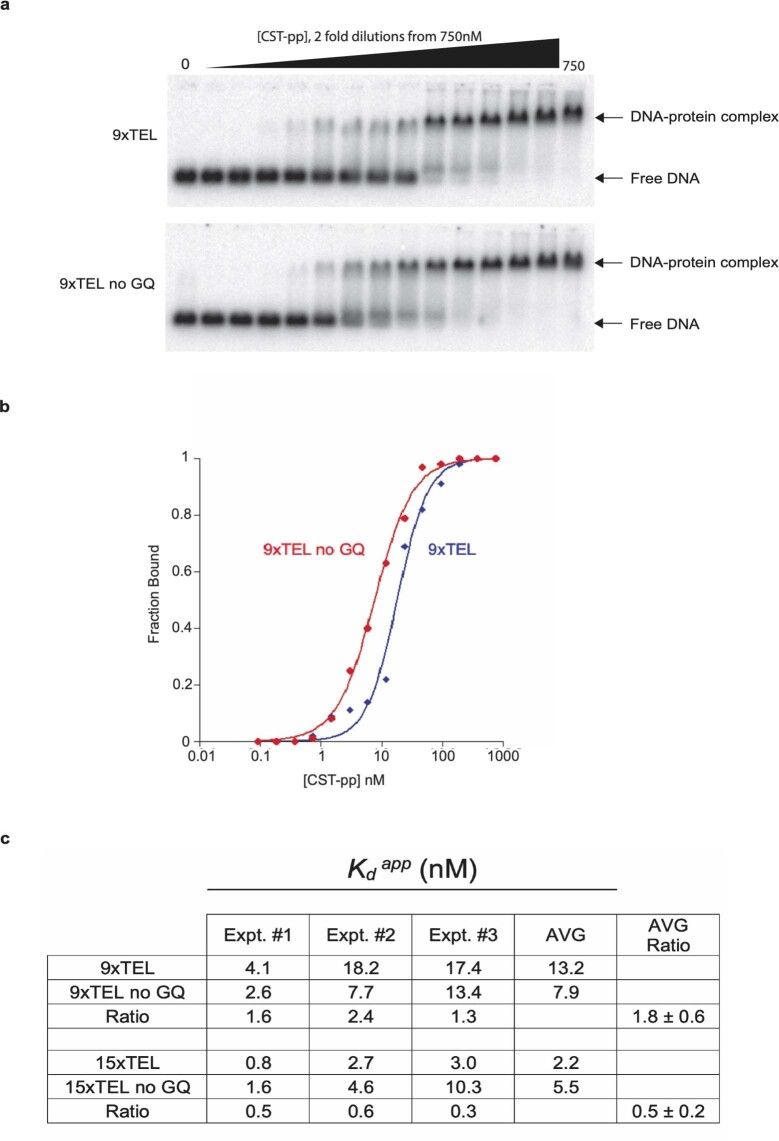
CST–Polα-primase binds similarly to GQ-forming and non-GQ-forming template DNAs. **a**, Typical EMSA measuring binding of labelled template DNA by CST–Polα-primase. **b**, Quantification of fraction bound = DNA-protein complex/(free DNA + DNA-protein complex) as a function of CST–Polα-primase concentration. Data points were fit to a binding equation where the Hill coefficient was allowed to vary to give best fit. **c**, Summary of apparent *K*_d_ values obtained from 12 independent experiments similar to those shown in **a** and **b**. The better binding of 9xTEL-noGQ relative to 9xTEL changed with the 15xTEL series, where 15xTEL bound better than 15xTEL-noGQ. This difference was not anticipated and is not understood, but it was reproducible; in any case, the differences in binding affinity between GQ and noGQ templates are small, not exceeding two-fold. For gel source data, see Supplementary Fig. 1 Source Data

Difficulties in replicating telomeres in vivo have been ascribed in part to GQ structure formation. Thus, we were surprised that the TEL templates functioned so well in the 50 mM KCl, 75 mM NaCl buffer used in the reactions of Figs. 1 and 2a, conditions known to stabilize GQs. To test whether disrupting GQ formation would affect C-strand synthesis, we retained 3×TEL as an origin of replication and then mutated the remaining TEL repeats from TTAGGG to TGAGTG. Breaking up the consecutive G’s prevented GQ formation (Extended Data Fig. 7), while the mutant sequence still bound CST–Polα–primase with an affinity within two-fold of that for the corresponding telomeric sequence (Extended Data Fig. 8). As shown in Fig. 3a, preventing GQ formation in the template greatly increased C-strand synthesis for both the 9×TEL and 15×TEL templates (7.0- ± 1-fold increase, mean ± range of values, *n* = 4). The products still have a hexameric repeat pattern, because the mutant sequence provides a CST-binding site every 6 nt. Under slightly different conditions where the only salt was 100 mM KCl, the templates without GQs (noGQ templates) again showed a large increase in activity: 10.5- ± 1.0-fold for 9×TEL-noGQ and 12.9- ± 0.7-fold for 15×TEL-noGQ (means ± ranges for *n* = 2; compare lane 12 to 15 and lane 18 to 21 in Fig. 3b).

To validate the inhibitory effect of GQs for the unmutated TEL templates, we relied on the cation specificity of GQ formation. That is, GQ folding is highly stabilized by K^+^, which fits in the central cavity of an intramolecular quadruplex and stabilizes the partial negative charges on the carbonyl oxygens of the four guanines in each G-quartet^28,29^. GQs are less stabilized by Na^+^ (ref. ^30^) and not stabilized by Li^+^. We first determined whether changing the cation would affect the intrinsic activity of CST–Polα–primase by using the 3×TEL-dT_72_ template, which cannot form intramolecular GQs because it lacks four tracts of guanine. Li^+^ reduced activity by 3.4- ± 0.4-fold relative to that with K^+^ (mean ± range, *n* = 2) (Fig. 3b). A similar decrease was seen with the 9×TEL-noGQ and 15×TEL-noGQ templates (3.8- ± 1.0-fold, *n* = 4). By contrast, the GQ-forming templates (9×TEL, 15×TEL, 25×TEL and 30×TEL) showed a 2.9-fold increase in C-strand synthesis in Li^+^ relative to K^+^ (see Fig. 3c for statistical details). Correcting for the 3.4-fold-lower activity of CST–Polα–primase in Li^+^ relative to K^+^, we conclude that substituting Li^+^ for K^+^ resulted in a 10-fold increase in activity for the GQ-forming templates. Substituting Na^+^ for K^+^ made no difference for the noGQ templates but gave a 3.8-fold increase in C-strand synthesis for the GQ-forming templates (Fig. 3c). Thus, CST–Polα–primase copies telomeric templates despite substantial inhibition by GQs.

## CST activation of Polα–primase

**Extended Data Fig. 9:**
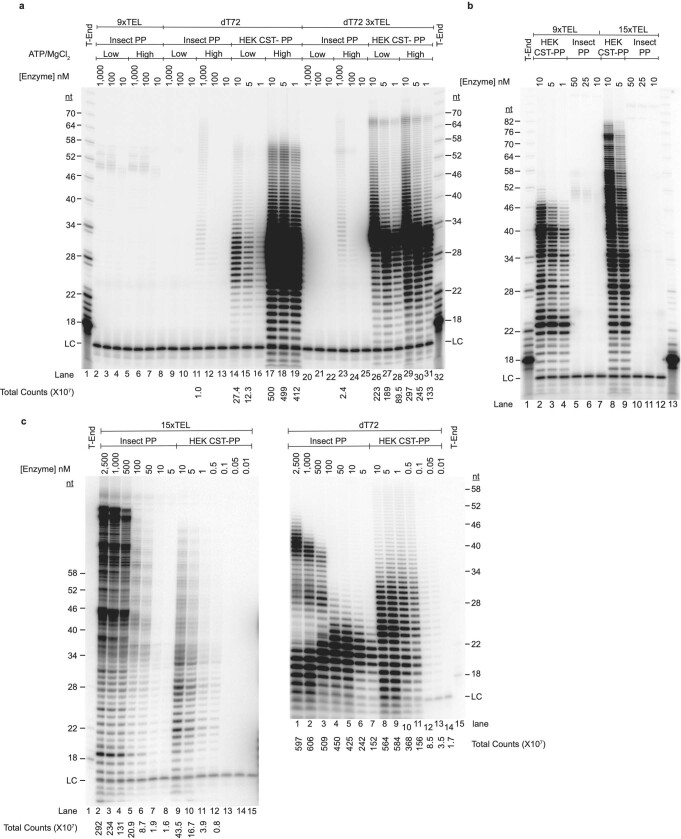
CST–Polα-primase purified from HEK cells is much more active on telomeric DNA templates than recombinant human Polα-primase purified from insect cells. **a**, Under two reaction conditions (low, 0.2 mM rATP and 1 mM MgCl_2_; high, 1.0 mM rATP and 2 mM MgCl_2_), CST–Polα-primase purified from HEK cells (*HEK CST-PP*) is more than 10,000x more reactive than human Polα-primase purified from insect cells (*Insect PP*). For example, compare 1000 nM Insect PP with 1 nM HEK CST-PP under the same reaction conditions: lanes 11 and 19, lanes 20 and 28, lanes 23 and 31. Phosphorimager counts are given for those lanes that had a signal above background. **b**, The extremely large difference in activity between CST-PP and PP also pertains to the 9xTEL and 15xTEL templates using the 'high' reaction conditions of **a** (1.0 mM rATP and 2 mM MgCl_2_). In both **a** and **b**, template DNA was 100 nM. **c**, Reactions under the conditions of ref. ^38^, with 50-fold higher DNA template concentrations (5 µM). For the 15xTEL template, the difference in activity was greater than 10-fold; compare the two 10 nM reactions, lanes 7 and 9; or 100 nM Insect PP with 10 nM HEK CST-PP, lanes 5 and 9; or 50 nM Insect PP with 5 nM HEK CST-PP, lanes 6 and 10. However, the processivity of the two enzymes was similar; compare lanes 5 and 6 with lanes 9 and 10. (The high-concentration Insect PP points in lanes 2–4 show re-priming, not high processivity.) For the dT72 template, there was very little difference in activity under these conditions; compare the two 10 nM reactions, lanes 6 and 8, or the two 5 nM reactions, lanes 7 and 9. Note that the HEK CST-PP is somewhat more processive than the Insect PP; compare dT72 lanes 8-11 with lanes 4-7. (The high-concentration Insect PP points (lanes 1-3) show re-priming, not high processivity.) For this entire figure, note that the HEK CST-PP concentrations are based on the concentration of CST, so the concentration of PP in these preparations is 5-fold lower (see Extended Data Fig. 1). Thus, in terms of PP, the large activity advantage of CST-PP indicated above is actually 5-fold larger. Each gel contains a telomerase ladder (T-End) as markers. Each lane contains the same loading control (LC), a labelled 16 nt DNA. For gel source data, see Supplementary Fig. 1.

**Extended Data Fig. 10:**
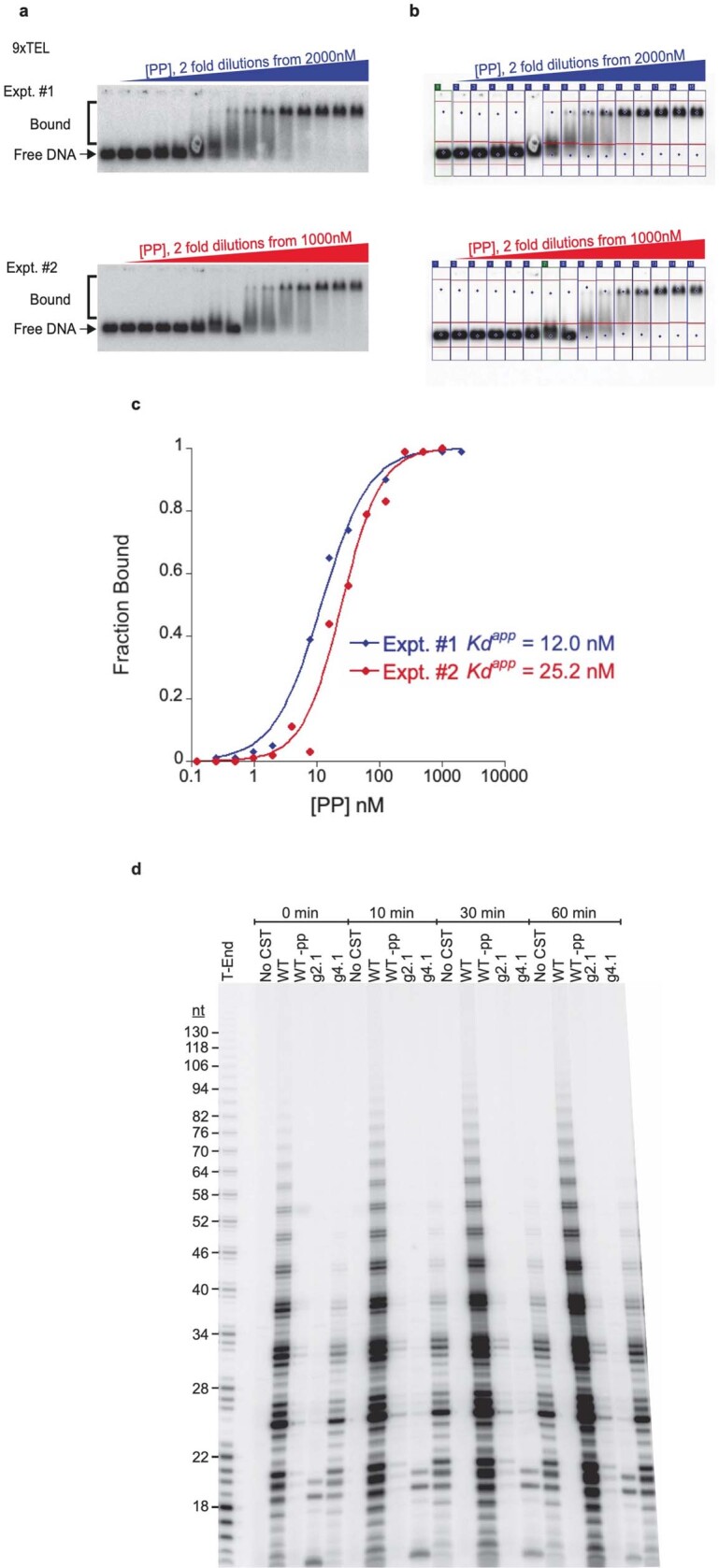
The lower activity of recombinant Polα-primase cannot be explained entirely by weaker binding to the ssDNA template and reconstitution of telomere end-replication. **a**, Two EMSA experiments measuring binding of Polα-primase purified from insect cells (Insect PP in Extended Data Fig. 1) with labelled 9xTEL DNA. **b**, Because these DNA-PP complexes dissociate during electrophoresis, the definition we used for free and bound DNA is shown here by the red horizontal lines. The bound species are taken to include both the fully bound complex and the smear of counts between bound and free DNA. **c**, Quantification of fraction bound as a function of Polα-primase concentration, performed as in Extended Data Fig. 8b. Although the apparent *K*_d_ values determined here for Insect cell PP are similar to those determined in Extended Data Fig. 8 for HEK cell CST-PP, recall that CST-PP is only 21% PP. Thus, in terms of PP binding, the CST-PP has 5-fold higher affinity. **d**, Reconstitution of complete telomere end-replication. C-strands were labelled with α-^32^P-dCTP. Time between initiation of telomerase reaction and addition of CST–Polα-primase is indicated at top. CST–Polα-primase is present at 75 nM. WT-pp is CST largely depleted of Polα-primase by conducting IP purification at high (300 mM) salt concentration. g2.1 is a DNA-binding defective mutant of CST, and g4.1 is a control mutant of CST that retains DNA binding but is present at only one-third the concentration as WT CST owing to lower yield during purification. For gel source data, see Supplementary Fig. 1 Source Data

To quantify the contribution of CST to Polα-primase activity, we compared CST–Polα–primase assembled in human cells with recombinant human Polα–primase overexpressed in insect cells. Notably, the CST-containing enzyme was more than 10,000-fold more active under our standard reaction conditions (Extended Data Fig. 9a,b). This large activation is in agreement with the initial discovery of CST as a replication accessory factor^4,5^. With high concentrations of enzyme and template DNA, conditions that overcome weak binding interactions, the CST-containing enzyme was still more than 10-fold more active than the recombinant Polα–primase (Extended Data Fig. 9c). (The recombinant Polα–primase differs from our human-cell enzyme in that it is assembled in insect cells and is missing the post-translational modifications of the endogenous enzyme; however, previous work indicates that these differences may not contribute much to the divergent activities of the two enzyme preparations^31^. Instead, the presence or absence of CST is likely to be the main difference). Furthermore, the affinity of the recombinant Polα–primase for DNA template was not much different than that of CST–Polα–primase, although the complex without CST dissociated during electrophoresis, suggesting a faster off rate (Extended Data Fig. 10a–c). Taken together, our results suggest that CST does more than simply promote binding of the DNA template to Polα–primase but also activates it for optimal primase activity. These functional conclusions are in excellent agreement with conclusions based on the recent cryo-EM structure^32^.

## Complete telomere end replication

Having studied the ability of CST–Polα–primase to synthesize C-strands when provided with synthetic telomeric templates, we attempted to reconstitute G-strand and C-strand synthesis in a single reaction. The mixtures contained 3×TEL as a primer for telomerase; notably, this primer is too short to give reaction products with CST–Polα–primase (Extended Data Fig. 2a,b), so CST–Polα–primase will be able to make C-strands only if telomerase extends 3×TEL to produce longer templates.

**Fig. 4:**
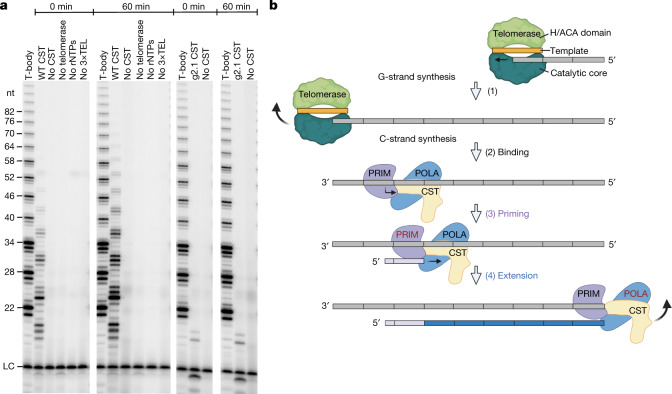
Reconstitution of complete telomere end replication. **a**, Telomerase products are unlabelled (except in the T-body marker lanes), and C-strands are labelled with [α-^32^P]dCTP. Time between initiation of the telomerase reaction and addition of 57 nM CST–Polα–primase (WT CST) is indicated at top. Control lanes are reactions identical to WT CST except containing no CST, no telomerase, no ribonucleotides (rNTPs) or no 3×TEL primer for telomerase. In the two right-hand sets of lanes, 57 nM g2.1 CST, a DNA-binding-defective mutant of CST, is substituted for WT CST. The uncropped gel is shown in Supplementary Fig. 1, and an experiment with intermediate time points is shown in Extended Data Fig. 10d. **b**, Model for reconstituted telomere replication. (1) Telomerase (RNA template in orange) binds to the 3×TEL DNA primer (grey) and extends it with telomeric repeats (grey rectangles). (2) Telomerase dissociates, and CST–Polα–primase binds to telomeric repeats and begins RNA primer synthesis with ATP. (3) Primase synthesizes RNA primer (~8 nt). (4) Template–primer pair is handed off to Polα, which catalyses C-strand DNA synthesis (blue bar). The continued presence of CST during extension is unknown.

As shown in Fig. 4a and Extended Data Fig. 10d, a robust ladder of C-strand products was formed even when telomerase and CST–Polα–primase were added simultaneously (0 min) and increased when the telomerase reaction was given a head start (60 min). The reactions were dependent on addition of CST–Polα–primase, telomerase, ribonucleotides and the 3×TEL primer for telomerase. Furthermore, the DNA-binding-defective g2.1 mutant had little activity. Thus, coupled G-strand and C-strand synthesis has been reconstituted in a reaction containing only telomerase, a 3×TEL DNA primer, CST–Polα–primase, ribonucleotides and deoxynucleotides.

## Discussion

The telomeric ssDNA overhang is a primitive replicon in that its replication does not involve a replication fork and requires only a limited number of macromolecules. As shown here, only two enzymatic components are required: the telomerase ribonucleoprotein complex and CST–Polα–primase. Furthermore, any pair of TTAGGG repeats can act as an ‘origin of replication’ or, more accurately, an origin of RNA priming and C-strand DNA synthesis. Note that the reaction products resemble a natural telomere, with double-stranded telomeric repeats and a 3′ overhang of the G-strand, the length of which depends on where CST–Polα-primase binds to initiate synthesis. The shortest 3′ overhangs produced in this system would need to be either extended further by telomerase or subjected to C-strand resection to give the 100- to 300-nt G overhangs seen in vivo^33^. Note that our data do not distinguish whether telomerase and CST–Polα–primase act independently (as modelled in Fig. 4b) or in a synergistic manner^20^.

To better approximate a natural telomere, we tested templates with a telomeric ssDNA–dsDNA junction and with the POT1–TPP1N telomeric protein bound. We found that C-strand synthesis terminated when it encountered dsDNA. Replication was neither enhanced nor inhibited by POT1–TPP1N except at high concentrations, where the observed inhibition was probably due to the template DNA being coated with protein.  We might have expected that this shelterin complex would affect CST–Polα–primase action, given that human POT1 and TPP1, as well as mouse POT1b, have been shown to bind CST^10,33^. Furthermore, a recent cryo-EM structure of *Tetrahymena* telomerase bound to CST–Polα–primase revealed an interaction between *Tetrahymena* p50 (an orthologue of human TPP1) and CST^34^. Given that we saw no impact on C-strand synthesis with stoichiometric POT1–TPP1N, it remains to be seen whether POT1–CST or TPP1–CST interactions that may occur in the human system affect C-strand synthesis.

Previously, CST was known to have two functions in telomere replication: termination of telomerase extension of the G-strand^10,11^ and recruitment of Polα–primase to begin replication of the C-strand^4,12,13^. We now find that CST has additional roles: it positions bound primase at telomeric repeats and activates it for primer synthesis. Remarkably, the mutants of CST that are defective in ssDNA binding have reduced ability to deposit their Polα–primase, and replication is substantially impaired. Thus, the reconstituted replication system now makes it clear why the partnership between CST and Polα–primase is so important to telomere replication. The fact that CST binds well to the telomeric repeat sequence allows it to position primase for initiation and then possibly move along the telomeric DNA template with Polα.

Numerous studies have found that telomeric DNA presents difficulties for replication, including problems with replication fork stalling (reviewed in ref. ^35^). Replication of both the dsDNA and ssDNA regions of telomeres could contribute to these difficulties. GQ structure formation has been suggested as a potential contributor, but this is difficult to test in vivo. Now, with our reconstituted biochemical system, we show that GQ formation in the G-strand template is, in fact, a barrier to telomeric C-strand synthesis; preventing GQ formation either by mutating the telomeric repeat sequence or by replacing K^+^ with Li^+^ greatly improves C-strand synthesis. Nevertheless, the telomeric repeats serve as an effective template owing to their tight binding to CST, which can also melt GQ structures^36^. This results in the telomeric DNA being a much better template than poly(dT), which has the advantage of being unstructured but has little affinity for CST.

This work also has implications for the mechanism by which CST restarts stalled replication forks across the genome. We find that even short G-rich sequences can bind CST sufficiently to promote Polα–primase action, and we suggest that most stalled replication forks would present such a sequence. Indeed, if a replication fork stalled because of GQ structure formation, it would necessarily have at least four G tracts and thus would recruit CST–Polα–primase in the same manner we have observed in the case of telomeric ssDNA.

Because telomere maintenance is required for cancer cell immortality, telomere end replication components may represent targets with specificity for tumour cells over normal cells, especially for short-telomere cancers. In support of this idea, the three genes for the CST components rose to the very top in CRISPR–Cas9 screens for genes important for growth of cancer cell lines with short telomeres^37^. On the basis of the work presented here, the ssDNA-binding site in the CTC1 subunit^19^ or Polα–primase-binding sites in all three CST subunits^32,38^ seem particularly attractive targets for interfering with telomere end replication.

## Methods

### Oligonucleotides

DNA oligonucleotides were purchased from Integrated DNA Technologies (IDT) and resuspended in water. Their concentrations were then measured by determining UV spectra (NanoDrop One, Thermo Fisher Scientific) using extinction coefficients provided by IDT. DNA oligonucleotides are named for the number of consecutive telomeric repeats (for example, 9×TEL is (TTAGGG)_9_) or mutated telomeric repeats (for example, 9×TEL-noGQ is (TGAGTG)_9_). Chimeric template sequences are named in the 5′-to-3′ polarity with respect to C-strand synthesis, which is therefore 3′ to 5′ with respect to the template (for example, 3×TEL-dT_72_ is a 90-nt oligonucleotide with the sequence 3′-GGGATTGGGATTGGGATT-T_72_-5′).

### Expression and purification of CST–Polα–primase in human cultured cells

HEK239T cells (CRL-1573, ATCC) were authenticated by ATCC using the COI assay for species determination, STR analysis (PCR based) and human pathogenic virus testing (PCR-based assay for HIV, hepatitis B virus, human papillomavirus, Epstein–Barr virus and cytomegalovirus); they were tested for mycoplasma contamination bimonthly and found to be negative. *CTC1* cDNA (MGC, 133331) with an N-terminal 3×FLAG tag, *STN1* cDNA (MGC, 2472) with an N-terminal Myc tag and *TEN1* cDNA (MGC, 54300) with an N-terminal HA tag were each cloned into the pcDNA mammalian expression vector (V79020, Thermo Fisher Scientific). The three plasmids were transfected into HEK293T cells at a 1:1:1 molar ratio using Lipofectamine 2000 (11668019, Thermo Fisher Scientific). The cells were further grown in DMEM supplemented with 2 mM l-glutamine, 1% penicillin-streptomycin and 10% FBS for 24 h after transfection (typically three-fold expansion) and then collected. Protein purification proceeded as described^11^ and involved successive anti-FLAG and anti-HA immunopurifications. The purity of CST complexes was verified with SDS–PAGE using a silver staining kit (24612, Thermo Fisher Scientific). To prepare CST stripped of Polα-primase, the NaCl concentration in the wash and elution buffers was increased from 150 mM to 300 mM. The presence of CST subunits and Polα–primase subunits in the purified complexes was determined by western blotting using the antibodies listed^11^. POLA1 consistently appeared as a doublet, probably owing to processing between Lys123 and Lys124 producing a stable 165-kDa species^39^; both species were included in the quantification of POLA1. CST protein concentrations were determined by western blot analysis with anti-STN1 antibody diluted 1:1,000 (NBP2-01006, Novus Biologicals) using a serial dilution of the HEK293T cell CST protein preparation and a standard curve obtained by serial dilution of an insect cell-purified CST standard (see ref. ^11^ for further discussion).

### Expression and purification of POT1–TPP1N

POT1 and TPP1N (amino acids 87–334)^40^ were expressed in *Escherichia coli* BL21(DE3) cells. Purification included size exclusion chromatography as described in refs. ^41,42^.

### C-strand synthesis reactions

Unless indicated otherwise, the following were the standard reaction conditions. HEK293T cell CST–Polα–primase (25–50 nM CST) and ssDNA templates (50–100 nM) were incubated at 30 °C for 1 h in 50 mM Tris-HCl pH 8.0, 50 mM KCl, 75 mM NaCl, 2 mM MgCl_2_, 1 mM spermidine, 5 mM β-mercaptoethanol, 0.5 mM dATP, 0.5 mM dTTP, 0.33 μM [α-^32^P]dCTP (300 Ci mmol^–1^), 0.29 μM unlabelled dCTP and 0.2 mM of each rNTP. When labelling with dATP, the concentration of unlabelled dATP was decreased to 10 μM and 0.33 μM [α-^32^P]dATP (300 Ci mmol^–1^) was added to the reaction instead of 0.33 μM [α-^32^P]dCTP (3000 Ci mmol^–1^). When labelling with [γ-^32^P]ATP, the concentration of unlabelled ATP was lowered to 25 μM. After incubation at 30 °C, 100 μl of Stop Mix (3.6 M ammonium acetate, 0.2 mg ml^–1^ glycogen and a 16-nt ^32^P-labelled loading control) and 500 μl ethanol were added to each 20-μl reaction. After incubating at –80 °C for 30 min or more, the products were pelleted, washed with cold 70% ethanol, dried and then dissolved in 10 μl water plus 10 μl of 2× loading buffer (93% formamide, 30 mM EDTA, 0.1× TBE and 0.05% each of bromophenol blue and xylene cyanol). Samples (9 μl) were loaded on a sequencing-style 10% acrylamide, 7 M urea, 1× TBE gel. The bromophenol blue dye was run to the bottom of the gel (about 1.75 h). The gel was removed from the glass plate using Whatman 3-mm paper and then dried at 80 °C under vacuum for 15–30 min. Radiolabelled C-strand synthesis products were imaged on a Typhoon FLA9500 scanner (GE Lifesciences) and analysed by ImageQuant TL v.8.1.0.0 (GE Lifesciences). Unless indicated otherwise, CST–Polα–primase activity was determined by total counts per lane corrected for any variation in the loading control. In some cases, individual reaction products were quantified.

### Telomerase purification and reactions

Telomerase reactions were run to provide marker lanes for the C-strand synthesis reactions. Human telomerase expression and purification followed ref. ^43^. To produce body-labelled products, telomerase was incubated at 30 °C for 1 h with 100 nM unlabelled 3×TEL DNA primer in 50 mM Tris-HCl pH 8.0, 50 mM KCl, 75 mM NaCl, 2 mM MgCl_2_, 1 mM spermidine, 5 mM β-mercaptoethanol, 0.33 μM [α-^32^P]dGTP (3,000 Ci mmol^−1^), 2.9 μM unlabelled dGTP, 0.5 mM dATP and 0.5 mM TTP. To produce end-labelled products, the reactions were identical except that 5′-^32^P-end-labelled 3×TEL DNA was substituted for the unlabelled primer and radiolabelled dGTP was omitted. For shorter products (less than 30 nt), the body-labelled products have reduced gel mobility relative to the end-labelled products by 1 nt, because the latter products have an additional phosphate on their 5′ ends. For long products (greater than 60 nt), the mobilities of the two sets of products converge. Note that neither set of telomerase products provides perfect markers for the C-strand products because the latter initiate with a triphosphate; in addition, electrophoretic mobility is somewhat base sequence dependent. Thus, most of our size estimates are prefaced by the symbol ~ indicating an uncertainty of ±1 nt.

### Complete telomere replication reactions

G-strands were synthesized by incubating immunopurified human telomerase (2.0 nM) and 3×TEL DNA primer (20 nM) at 30 °C in 50 mM Tris-HCl pH 8.0, 50 mM KCl, 75 mM NaCl, 2 mM MgCl_2_, 1 mM spermidine, 5 mM β-mercaptoethanol, 0.5 mM dATP, 0.5 mM dTTP, 3.3 μM dGTP, 2.9 μM dCTP, 0.33 μM [α-^32^P]dCTP and 0.2 mM of each rNTP. At the indicated times between 0 and 60 min, 19 μl was transferred to a tube containing 1 μl CST (WT, WT-pp or g2.1; 57 or 75 nM final concentration, as indicated in the figure legends) to initiate the synthesis of C-strands. WT and g2.1 include Polα–primase, whereas WT-pp is CST with most of the Polα–primase removed by a high-salt wash^11^. After an additional 1-h incubation at 30 °C, 100 μl of Stop Mix (3.6 M ammonium acetate and 0.2 mg ml^−1^ glycogen) and 500 μl ethanol were added to the 20-μl reaction. Ethanol precipitation, gel electrophoresis and phosphorimager scanning then proceeded as described above for C-strand synthesis reactions.

### Native gel electrophoresis to assess folding of ssDNA

ssDNA oligonucleotides were 5′-end labelled with [γ-^32^P]ATP (PerkinElmer) and run through a G-25 spin column (Roche). A 10% polyacrylamide gel (0.4-mm thickness) was poured using 0.5× TBE buffer containing 100 mM KCl, LiCl or NaCl. Gels were prerun at ∽80 V at a maximum of 6 W, using 0.5× TBE running buffer containing 100 mM KCl, LiCl or NaCl for ∽2.5 h in a 30 °C warm room. A gel apparatus with a heat exchanger connected to a 30 °C circulating water bath was used. The DNA was prepared in a mixture having final concentrations of 100 mM salt (KCl, LiCl or NaCl), 50 mM Tris-HCl pH 8.0, 1 mM MgCl_2_, 5 mM β-mercaptoethanol, 1 mM spermidine and loading dye. Samples were incubated for 15 min at 30 °C. They were then loaded on the gel and electrophoresis continued until the bromophenol blue dye reached 10.5 cm from the bottom of the well (∽5–6 h).

### Electrophoresis mobility shift assay

The method followed that of ref. ^11^. The oligonucleotides were 5′-end labelled with [γ-^32^P]ATP (NEG035C005MC, PerkinElmer) using phage T4 polynucleotide kinase (M0201L, NEB). Each binding reaction (10-μl sample volume) contained 500 counts per minute of radiolabelled DNA in binding buffer (20 mM HEPES-NaOH pH 8.0, 150 mM NaCl, 2 mM MgCl_2_, 0.2 mM EGTA, 0.1% NP-40, 10% glycerol, 1 mM DTT) with or without CST added. The binding reactions were incubated on ice for 2 h before loading onto a 1× TBE, 0.7% SeaKem LE Agarose (50004, Lonza) agarose gel. Gel electrophoresis was performed in a cold room (4 °C) for 1.5 h at 6.6 V cm^–1^. The gels were dried on Hybond N+ (RPN303B, Cytiva Amersham) and two pieces of 3MM chromatography paper (3030917, Cytiva Whatman) at 80 °C for 1.25 h. They were then exposed to a phosphorimager screen overnight. The screen was imaged with a Typhoon FLA9500 scanner (GE Lifesciences). The fraction of the DNA bound *θ* was calculated by dividing the counts from the gel-shifted band(s) by the total counts per lane. The apparent dissociation constant, *K*_d,app_, was then determined by fitting the fraction bound (*θ*) values to the Hill equation, *θ* = *P*^*n*^/(*P*^*n*^ + *K*_d,app_^*n*^), where *P* is the protein concentration and *n* is the Hill coefficient.

### Experimental reproducibility

The number of independent repeats of each experiment is as follows (in all cases, the repeat experiments gave equivalent results). Replicates were as follows for the main-text figures: Fig. 1, two repeats exactly as shown, plus 10 independent side-by-side comparisons of 9×TEL and 15×TEL in other contexts; Fig. 2a, in addition to the data shown, two additional independent experiments compared WT CST, g2.1 CST and g3.1 CST each with no template, 3×TEL, 9×TEL and 15×TEL; Fig. 2b, two independent repeats, plus three additional side-by-side comparisons of dT_54_, 1×TEL-dT_54_ and 2×TEL-dT_54_, plus two independent repeats of 2×TEL-dT_18_; Fig. 3a,b, three independent repeats each; Fig. 4, five independent repeats. For the Extended Data figures, the replicates were as follows: Extended Data Fig. 1, repeated in its entirety two times; the silver-stained gels and western blots for CST subunits were performed for each of the four independent CST–Polα–primase purifications used in this study; Extended Data Fig. 2a, two independent repeats; Extended Data Fig. 2b, two independent repeats; Extended Data Fig. 2c, three independent repeats; Extended Data Fig. 2d, one repeat; Extended Data Fig. 2e, three independent repeats as shown; Extended Data Fig. 3, one repeat of the entire experiment and two additional repeats comparing the three templates at template DNA concentrations of 100 and 5,000 nM; Extended Data Fig. 4, one repeat of the entire experiment and 10 independent repeats comparing the two templates at single DNA concentrations; Extended Data Fig. 5a, one repeat; Extended Data Fig. 5b, two repeats; Extended Data Fig. 6, one repeat; reproducibility indicated by the multiple time points; Extended Data Fig. 7, two repeats each of the three experiments; Extended Data Fig. 8, three complete experiments of the four DNA templates, with 12 electrophoretic mobility shift gels in total; Extended Data Fig. 9, repeats are shown within the figure; Extended Data Fig. 10a–c, two repeats, both of which are shown; Extended Data Fig. 10d, five independent repeats.

### Model illustrations

Figures 1c, 2c and 4b were created with BioRender.com.

### Reporting summary

Further information on research design is available in the Nature Research Reporting Summary linked to this paper.

## Online content

Any methods, additional references, Nature Research reporting summaries, source data, extended data, supplementary information, acknowledgements, peer review information; details of author contributions and competing interests; and statements of data and code availability are available at 10.1038/s41586-022-04930-8.

## Supplementary information

Supplementary Figure 1 Uncropped gels for Figs. 1–4 and Extended Data Figs. 1–10.

Reporting Summary

## Data Availability

Primary data that are necessary to interpret, verify and extend the research in this article are provided in Supplementary Fig. 1, which includes uncropped versions of all gels and blots. Source data are provided with this paper.

## Source data

Source Data Figs. 1 and 2 and Extended Data Figs. 1, 3 and 4
Source Data Extended Data Fig. 2
Source Data Extended Data Figs. 8 and 10

## Extended data

are available for this paper at 10.1038/s41586-022-04930-8.
