# Supplementary material for: Reconstitution of a telomeric replicon organized by CST
Source: Nature. 2022 Jul 13;608(7924):819–25. doi: 10.1038/s41586-022-04930-8 (PMC9402439; doi:10.1038/s41586-022-04930-8)

nature portfolio

https://doi.org/10.1038/s41586-022-04930-8

Supplementary information

Reconstitution of a telomeric replicon organized by CST

In the format provided by the
authors and unedited

Figure 1a

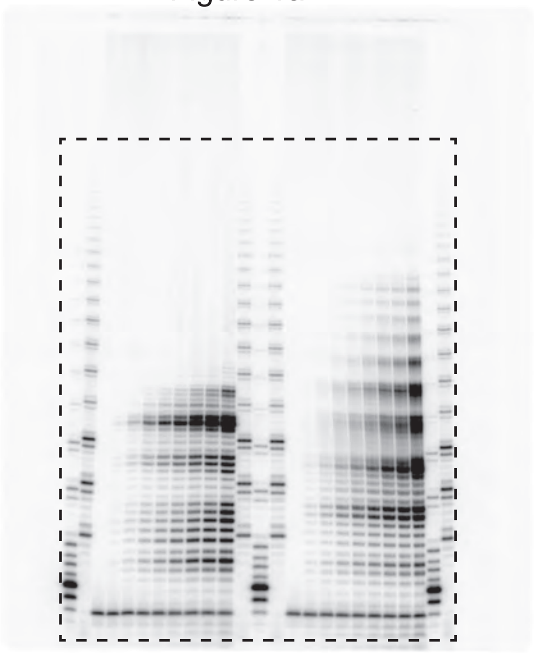

Figure 2a

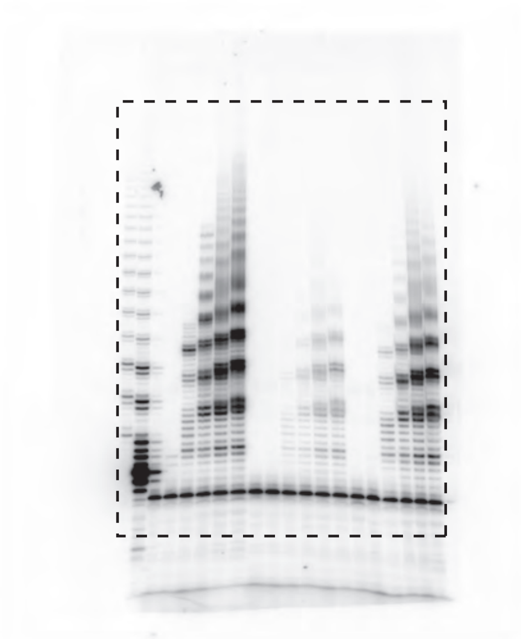

Figure 2b

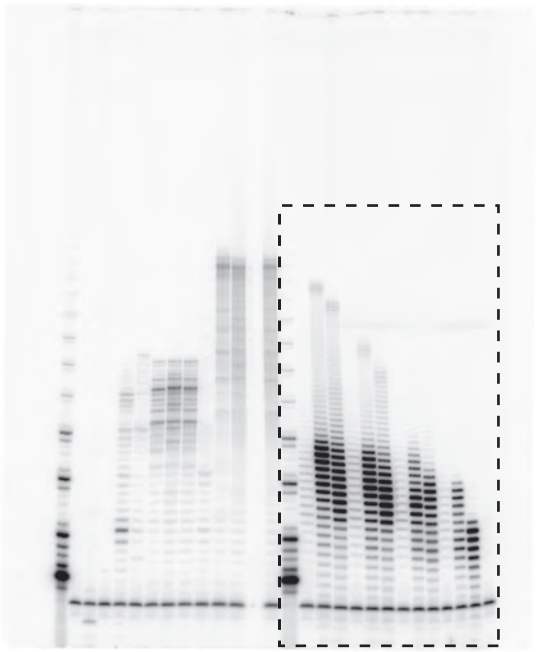

Figure 3a

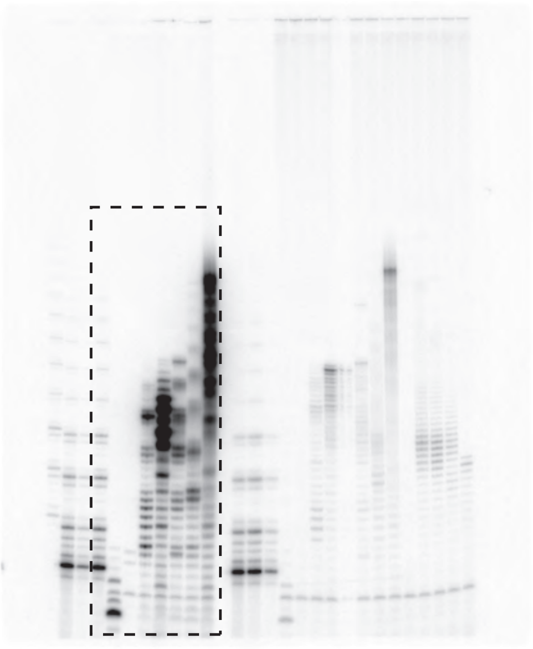

Figure 3b

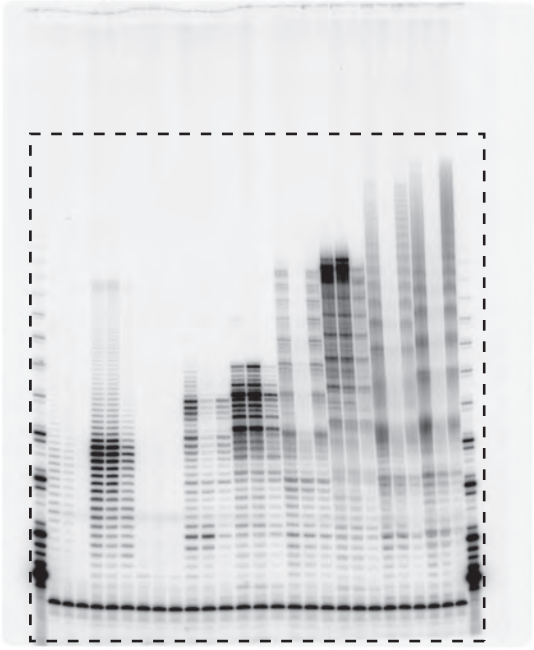

Figure 4a

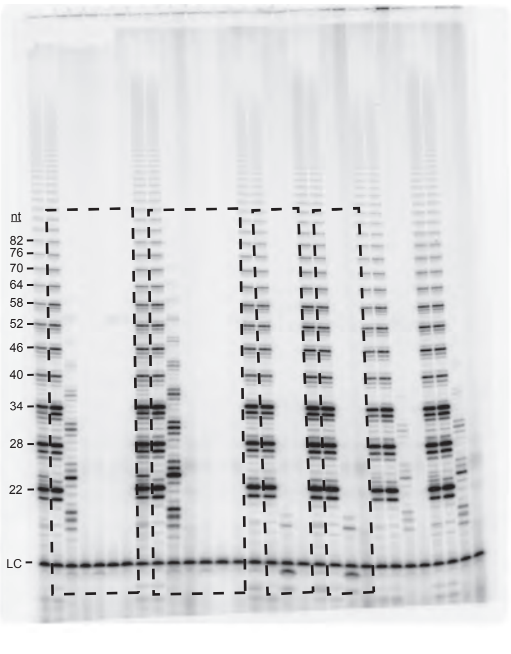

Extended Data Figure 1a

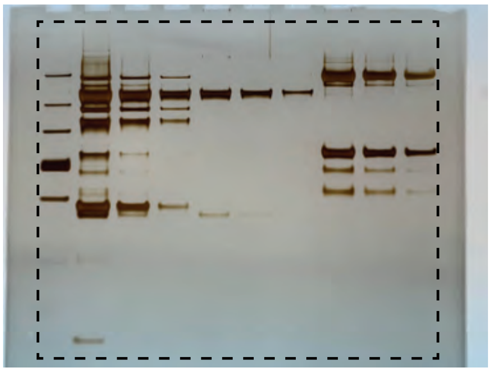

Extended Data Figure 1b

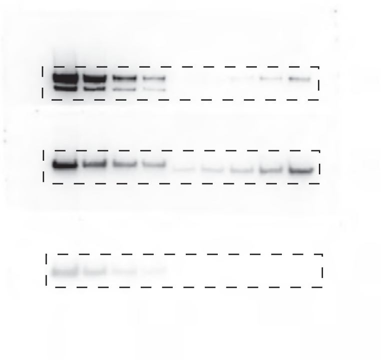

Extended Data Figure 1e

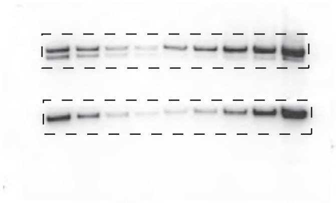

Extended Data Figure 2a

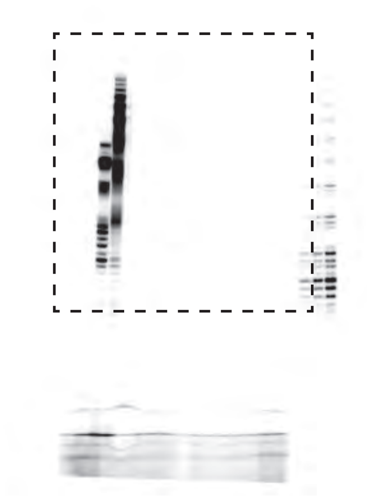

Extended Data Figure 2b

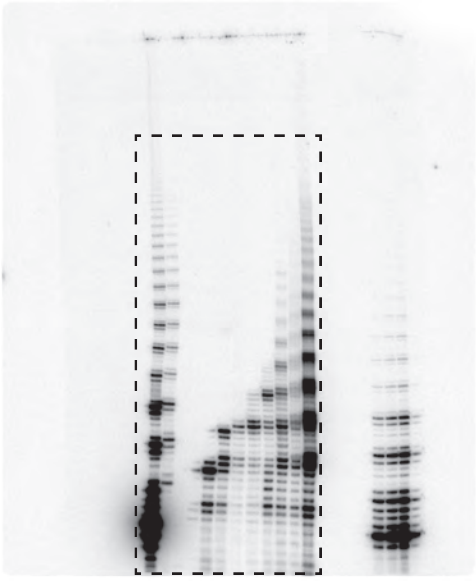

Extended Data Figure 2c

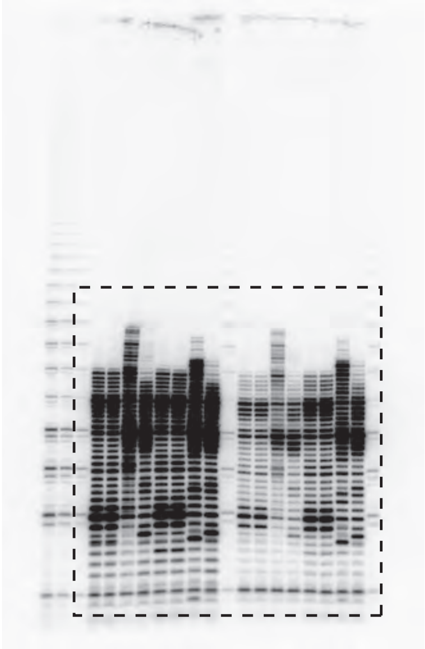

Extended Data Figure 2d

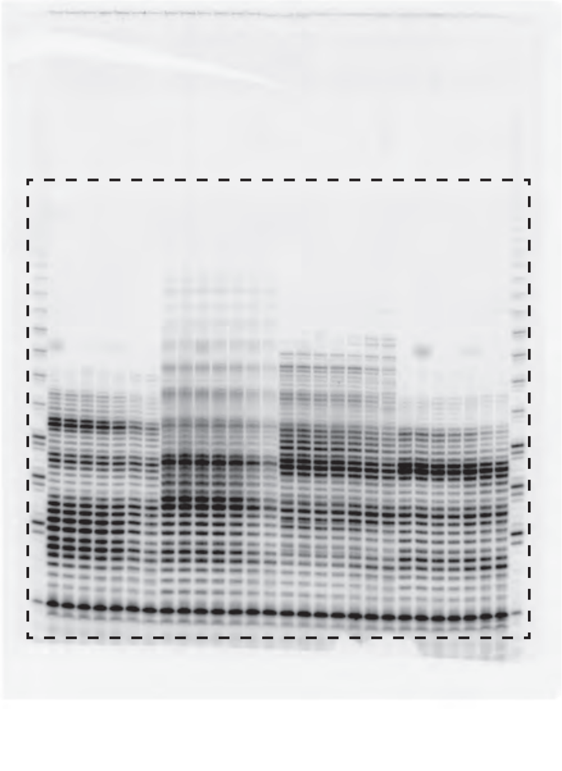

Extended Data Figure 2e

Experiment #1

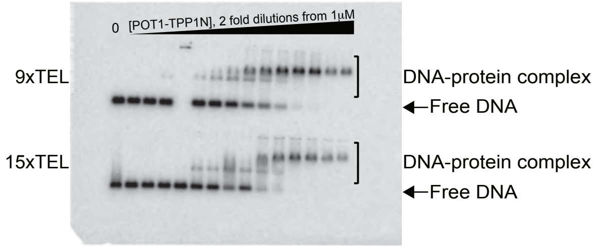

Experiment #2

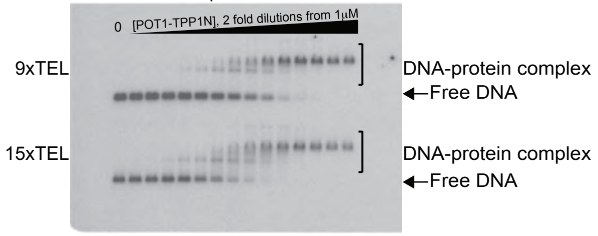

Experiment #3

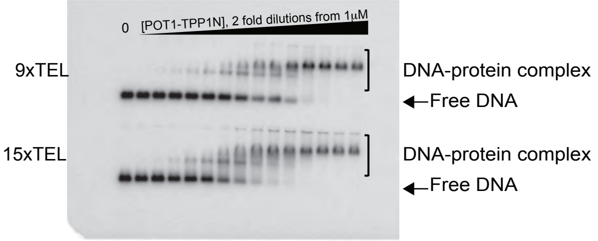

Extended Data Figure 3a

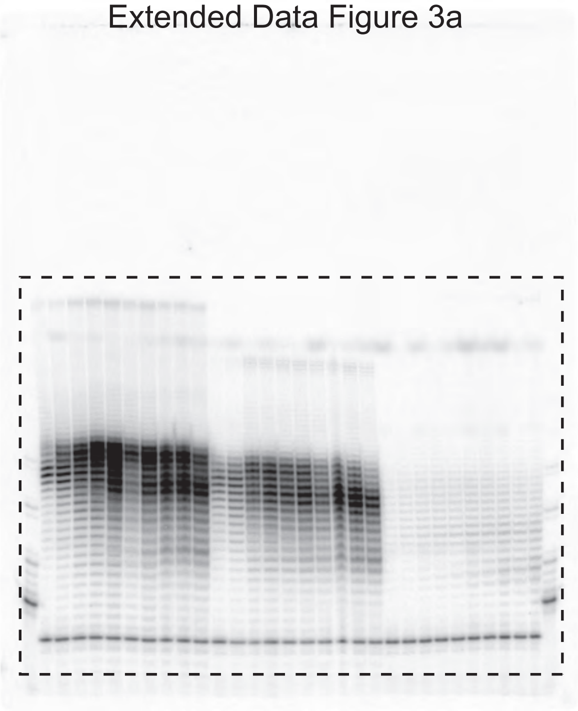

Extended Data Figure 4a

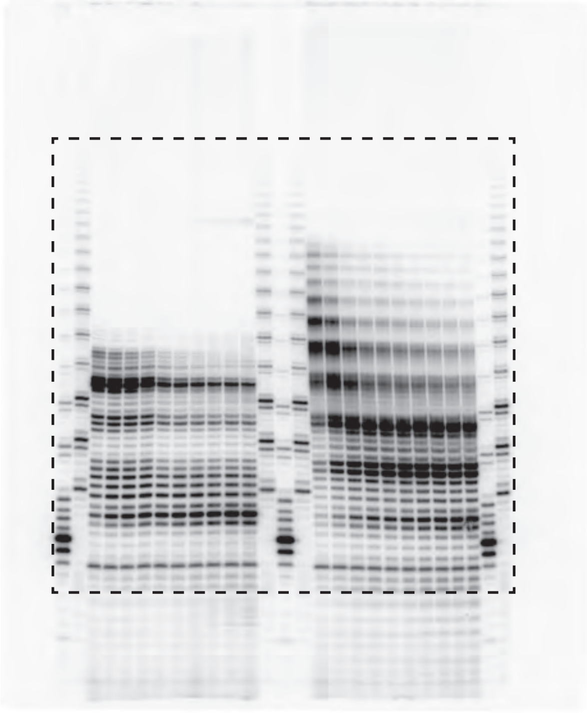

Extended Data Figure 5a

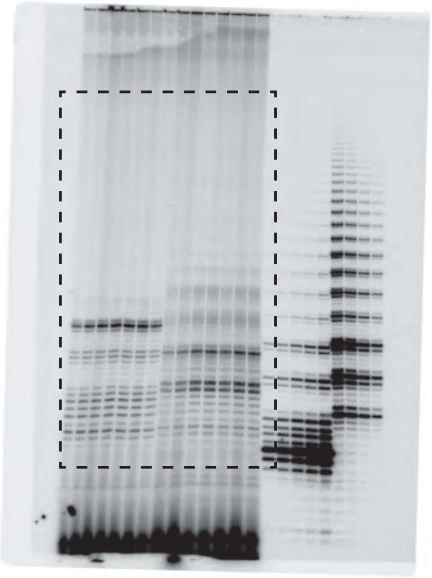

Extended Data Figure 5b

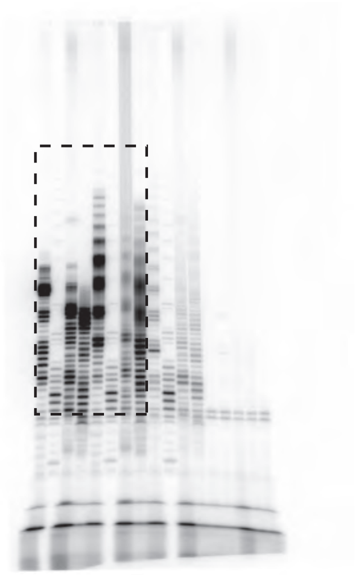

Extended Data Figure 6
4 day exposure

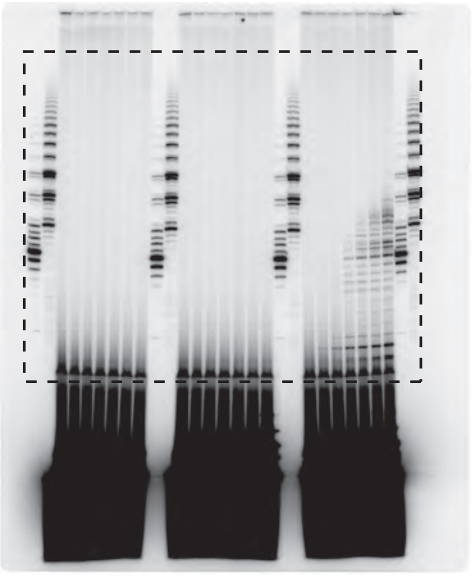

Extended Data Figure 6
4 hour exposure

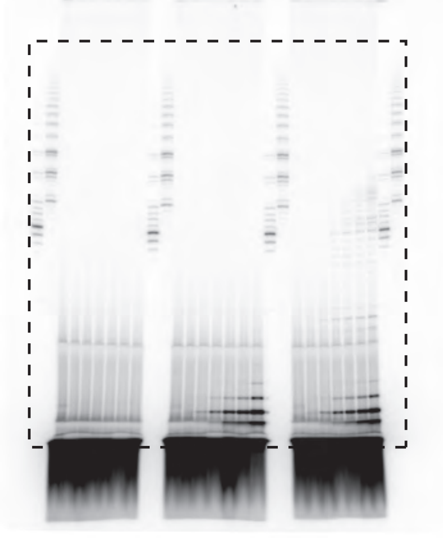

Extended Data Fig. 7

KCl

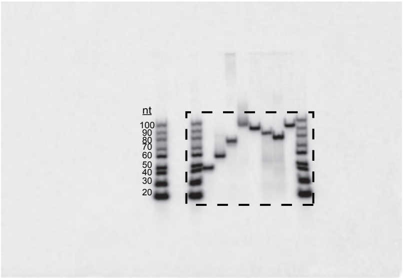

LiCl

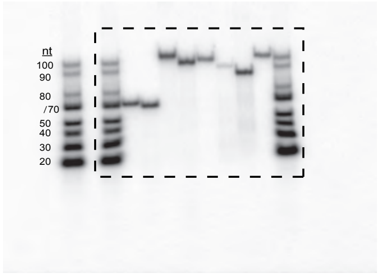

NaCl

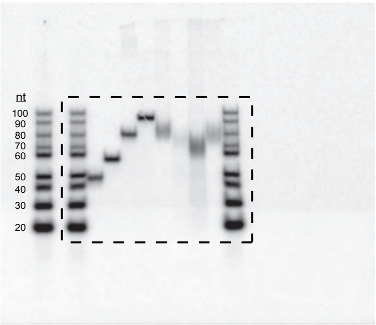

Extended Data Fig. 8

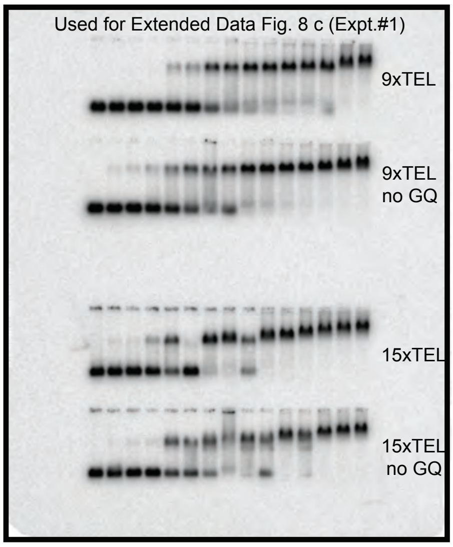

Extended Data Fig. 8

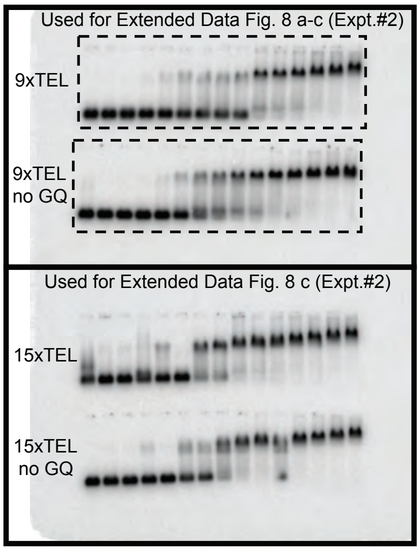

Extended Data Fig. 8

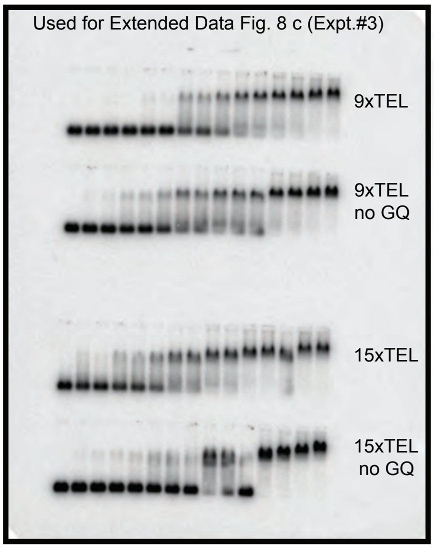

Extended Data Figure 9a

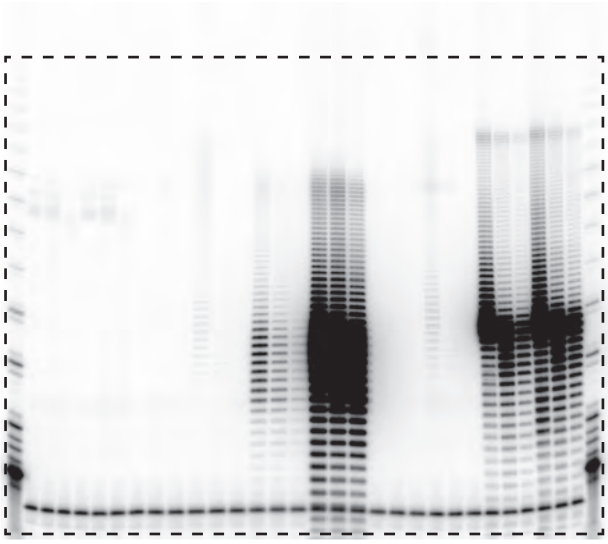

Extended Data Figure 9b

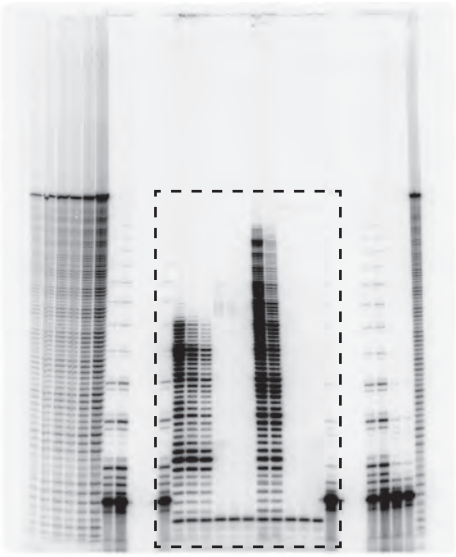

Extended Data Figure 9c

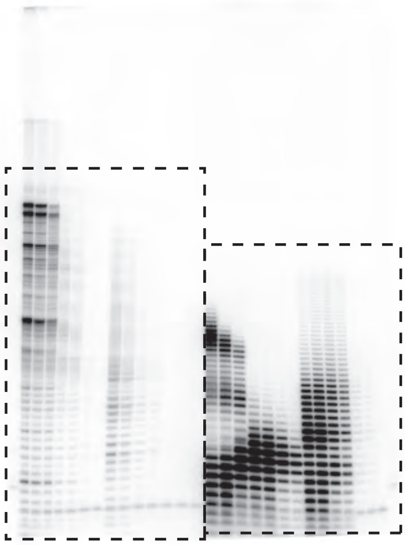

Extended Data Fig. 10 a-c

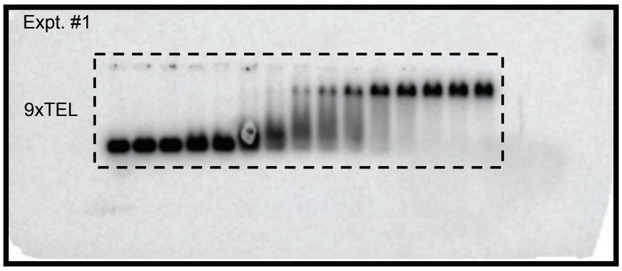

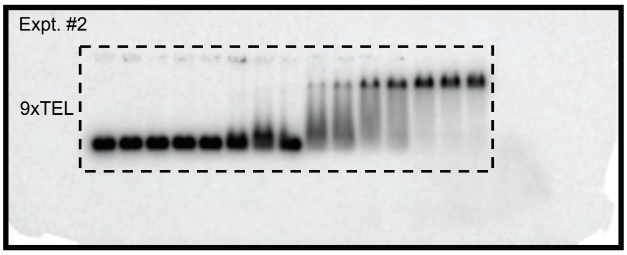

Extended Data Figure 10d

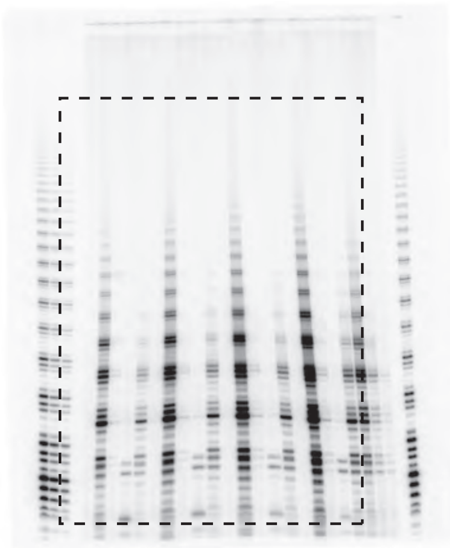

Supplement: Supplementary Figure 1 — Uncropped gels for Figs. 1–4 and Extended Data Figs. 1–10. [file 41586_2022_4930_MOESM1_ESM.pdf]
