# Supplementary material for: Reconstitution of a telomeric replicon organized by CST
Source: Nature. 2022 Jul 13;608(7924):819–25. doi: 10.1038/s41586-022-04930-8 (PMC9402439; doi:10.1038/s41586-022-04930-8)
Supplement: Supplementary file 2 — Reporting Summary [file 41586_2022_4930_MOESM2_ESM.pdf]

nature portfolio

Corresponding author(s): Thomas R. Cech

Last updated by author(s): May 18, 2022

Reporting Summary

Nature Portfolio wishes to improve the reproducibility of the work that we publish. This form provides structure for consistency and transparency in reporting. For further information on Nature Portfolio policies, see our Editorial Policies and the Editorial Policy Checklist.

Statistics

For all statistical analyses, confirm that the following items are present in the figure legend, table legend, main text, or Methods section.

n/a	Confirmed	
☐	☒	The exact sample size (*n*) for each experimental group/condition, given as a discrete number and unit of measurement
☐	☒	A statement on whether measurements were taken from distinct samples or whether the same sample was measured repeatedly
☐	☒	The statistical test(s) used AND whether they are one- or two-sided<br>*Only common tests should be described solely by name; describe more complex techniques in the Methods section.*
☐	☒	A description of all covariates tested
☒	☐	A description of any assumptions or corrections, such as tests of normality and adjustment for multiple comparisons
☐	☒	A full description of the statistical parameters including central tendency (e.g. means) or other basic estimates (e.g. regression coefficient) AND variation (e.g. standard deviation) or associated estimates of uncertainty (e.g. confidence intervals)
☐	☒	For null hypothesis testing, the test statistic (e.g. *F*, *t*, *r*) with confidence intervals, effect sizes, degrees of freedom and *P* value noted<br>*Give P values as exact values whenever suitable.*
☒	☐	For Bayesian analysis, information on the choice of priors and Markov chain Monte Carlo settings
☒	☐	For hierarchical and complex designs, identification of the appropriate level for tests and full reporting of outcomes
☒	☐	Estimates of effect sizes (e.g. Cohen's *d*, Pearson's *r*), indicating how they were calculated

Our web collection on statistics for biologists contains articles on many of the points above.

Software and code

Policy information about availability of computer code

Data collection	None used.
Data analysis	Scans of replication products on gels were quantified by ImageQuant TL v8.1.0.0 (GE Lifesciences). BioRender.com is not Data analysis software, but rather a web-based program for drawing models, and it has no version number.

For manuscripts utilizing custom algorithms or software that are central to the research but not yet described in published literature, software must be made available to editors and reviewers. We strongly encourage code deposition in a community repository (e.g. GitHub). See the Nature Portfolio guidelines for submitting code & software for further information.

Data

Policy information about availability of data

All manuscripts must include a data availability statement. This statement should provide the following information, where applicable:

- Accession codes, unique identifiers, or web links for publicly available datasets
- A description of any restrictions on data availability
- For clinical datasets or third party data, please ensure that the statement adheres to our policy

Primary data that are necessary to interpret, verify and extend the research in this article are provided in Supplementary Fig. 1, which included uncropped versions of all gels and blots. Source data are provided with this paper.

Field-specific reporting

Please select the one below that is the best fit for your research. If you are not sure, read the appropriate sections before making your selection.

☒ Life sciences ☐ Behavioural & social sciences ☐ Ecological, evolutionary & environmental sciences

For a reference copy of the document with all sections, see nature.com/documents/nr-reporting-summary-flat.pdf

Life sciences study design

All studies must disclose on these points even when the disclosure is negative.

Sample size	Not applicable, because no animals were used, nor were multiple cell lines compared.
Data exclusions	In experiments involving concentration series, there was an occasional bad lane on a gel that was excluded from quantification. These are seen as omitted points in Source Data.
Replication	The number of independent, successful repeats of each experiment is now indicated in a new section of Methods entitled "Experimental reproducibility."
Randomization	None done. (All of our experiments are biochemical experiments performed with purified molecular reagents, so "randomization" and "experimental groups" are not applicable.)
Blinding	No blinding was done for the experiments presented.

Reporting for specific materials, systems and methods

We require information from authors about some types of materials, experimental systems and methods used in many studies. Here, indicate whether each material, system or method listed is relevant to your study. If you are not sure if a list item applies to your research, read the appropriate section before selecting a response.

Materials & experimental systems

n/a	Involved in the study	
☐	☒	Antibodies
☐	☒	Eukaryotic cell lines
☒	☐	Palaeontology and archaeology
☒	☐	Animals and other organisms
☒	☐	Human research participants
☒	☐	Clinical data
☒	☐	Dual use research of concern

Methods

n/a	Involved in the study	
☒	☐	ChIP-seq
☒	☐	Flow cytometry
☒	☐	MRI-based neuroimaging

Antibodies

Antibodies used	anti-STN1, Novus Biologicals, Centennial, CO, NBP2-01006, diluted 1:1000.
Validation	Manufacturer's website validates by Western blot, Immunofluorescence of cells transfected with the gene, and flow cytometry of expressing cells. Our own validation involved comparison on Western blot with CST purified to homogeneity from baculovirus-infected insect cells. Ref: Schuck PL, Ball LE, Stewart JA, The DNA-binding protein CST associates with the cohesin complex and promotes chromosome cohesion, The Journal of Biological Chemistry (2021).

Eukaryotic cell lines

Policy information about cell lines

Cell line source(s)	HEK293T, CRL-1573, ATCC, Manassas, VA
Authentication	The only authentication done by us was by cell morphology; ATCC is a reputable provider and they may have done additional authentication, but they have not responded to my request for details.
Mycoplasma contamination	Our HEK293T cells were tested bimonthly for mycoplasma contamination in our Tissue Culture Core Facility, and they were found to be negative throughout this project.
Commonly misidentified lines (See ICLAC register)	Although some HEK cell lines are listed in the register, our HEK293T cells were obtained from ATCC and are not misidentified.
